# A Spatio-Temporal Model of Notch Signalling in the Zebrafish Segmentation Clock: Conditions for Synchronised Oscillatory Dynamics

**DOI:** 10.1371/journal.pone.0016980

**Published:** 2011-02-28

**Authors:** Alan J. Terry, Marc Sturrock, J. Kim Dale, Miguel Maroto, Mark A. J. Chaplain

**Affiliations:** 1 Division of Mathematics, University of Dundee, Dundee, United Kingdom; 2 Division of Cell and Developmental Biology, University of Dundee, Dundee, United Kingdom; University of Nottingham, United Kingdom

## Abstract

In the vertebrate embryo, tissue blocks called somites are laid down in head-to-tail succession, a process known as somitogenesis. Research into somitogenesis has been both experimental and mathematical. For zebrafish, there is experimental evidence for oscillatory gene expression in cells in the presomitic mesoderm (PSM) as well as evidence that Notch signalling synchronises the oscillations in neighbouring PSM cells. A biological mechanism has previously been proposed to explain these phenomena. Here we have converted this mechanism into a mathematical model of partial differential equations in which the nuclear and cytoplasmic diffusion of protein and mRNA molecules is explictly considered. By performing simulations, we have found ranges of values for the model parameters (such as diffusion and degradation rates) that yield oscillatory dynamics within PSM cells and that enable Notch signalling to synchronise the oscillations in two touching cells. Our model contains a Hill coefficient that measures the co-operativity between two proteins (Her1, Her7) and three genes (*her1*, *her7*, *deltaC*) which they inhibit. This coefficient appears to be bounded below by the requirement for oscillations in individual cells and bounded above by the requirement for synchronisation. Consistent with experimental data and a previous spatially non-explicit mathematical model, we have found that signalling can increase the average level of Her1 protein. Biological pattern formation would be impossible without a certain robustness to variety in cell shape and size; our results possess such robustness. Our spatially-explicit modelling approach, together with new imaging technologies that can measure intracellular protein diffusion rates, is likely to yield significant new insight into somitogenesis and other biological processes.

## Introduction

In the vertebrate embryo, tissue blocks called somites are laid down in head-to-tail succession [1]. This process is known as somitogenesis. The somites are laid down in pairs with one somite to either side of the central body axis. The number of somite pairs that are formed varies from species to species: zebrafish, chickens, mice, and corn snakes form 31, 53, 65, and 315 pairs respectively [2], [3]. Somites give rise to the musculo-skeletal segments of the neck, trunk, and tail [4]. Understanding the formation of somites is a highly active area of research [2]–[8].

**Figure 1:**
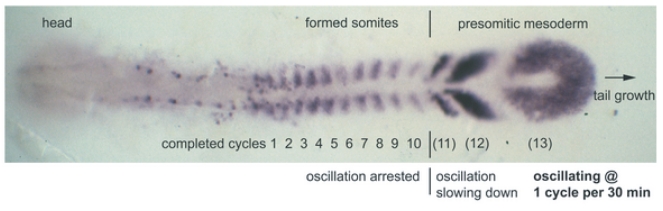
Top view micrograph of formed and forming somites in a zebrafish embryo. The embryo is at 10-somite stage, stained by in situ hybridization for *deltaC* mRNA. The *deltaC* gene exhibits oscillatory expression in the presomitic mesoderm (PSM). The oscillations are quickest in cells at the tail end (posterior) PSM. Cells in the posterior PSM enter the anterior PSM and then the somitic mesoderm (the region of formed somites) as the tail end of the embryo grows away from them. As the tail end grows it releases a Wnt and FGF signal. This gives rise to a morphogen gradient, which causes the oscillations to slow down in cells the further they are from the tail end. Cells cease oscillating altogether and form somites as they exit the anterior PSM. The spatio-temporal expression pattern of the *her1* and *her7* genes is very similar to the expression pattern for *deltaC*. Reproduced from figure 1a in [5] under the Creative Commons Attribution License.

Somites derive from cells in the presomitic mesoderm (PSM) at the tail end of the embryo (figure 1). Various experiments have revealed that cells in the PSM oscillate in their expression of certain genes that belong to the Notch signalling pathway [9]–[12]. These oscillations appear to be synchronised in neighbouring cells and, in zebrafish at least, this appears to be due to Notch signalling [1], [6], [13], [14]. The oscillations in a particular PSM cell slow down as the tail bud of the embryo grows away from it caudally. This is believed to be related to a morphogen gradient. Wnt and fibroblast growth factor (FGF) signalling molecules are produced at the tail end of the PSM. The further from this signalling source, the weaker the signal strength, and the slower the oscillations in PSM cells [2], [4], [15], [16]. At the point where the level of Wnt and FGF falls below some critical value, oscillations stop altogether and the cells form pairs of somites which bud off sequentially from the anterior PSM to form the somitic mesoderm (figure 1). Each somite has an antero-posterior polarity. The anterior portion of a somite expresses a different set of genes to the posterior portion [17]. The genes expressed in a somite depend upon the stage at which the PSM oscillations in gene expression were arrested [1].

Somitogenesis is often said to proceed by a “clock-and-wavefront” mechanism [18]–[20]. Here the term “clock” refers to the oscillatory gene expression in the PSM and the term “wavefront” refers to the moving interface between the PSM and the determined somite tissue. In one complete clock *cycle* a new pair of somites is formed, and this corresponds to the time taken for one complete oscillation of gene expression in the posterior PSM [1]. In that somites are *segmented* tissue blocks which form or are *determined* when they encounter the wavefront, the somitogenesis clock is frequently referred to as the segmentation clock and the wavefront as the determination wavefront.

Mathematical models of biological processes can yield insight that would be difficult to obtain by other means. Models of somitogenesis have been appearing for over 30 years, evolving in parallel with advances in experimental procedures and discoveries (see [21] and references therein). Mathematical models provide a theoretical framework for explaining observed phenomena and their predictions can guide experimentalists in devising new experiments. Indeed, the clock-and-wavefront mechanism for somitogenesis was originally proposed as a mathematical model and was based on minimal biochemical evidence [22]. It was only later that experimental evidence began to mount in its favour, allowing the finer details of the proposed mechanism to be updated [1], [6], [8], [23], [24]. Examples of mathematical models of somitogenesis include pattern formation models based on reaction-diffusion assumptions [25]–[30] or various other mechanisms [31]–[35] and cell-based models employing systems of ordinary differential equations (ODEs) [36]–[40] or delay differential equations (DDEs) [1], [6], [21], [41]–[45]. Many of these cell-based models attempt to capture the oscillations in gene expression in individual PSM cells, in some instances by artificial mathematical constructions. For example, in an ODE model for the self-repressing transcription factor Hes1 in mice, an unknown protein was introduced to encourage the system to oscillate [36]. However, by including delays for transcription and translation, it is possible to obtain oscillatory dynamics in simple models of self-repressing transcription factors without invoking the existence of unknown proteins [1], [6], [45].

Despite the growing number of mathematical models of somitogenesis, there seems to be a notable absence of a particular kind of model in the literature to date. Specifically, there are, to our knowledge, no models of somitogenesis that explicitly consider the movement of protein and mRNA molecules within a cell. Yet it is precisely the movement of molecules, and the molecular interactions thereby caused, that determine the dynamics within a cell. Indeed the importance of molecular movements in intracellular processes has been recognised in various studies not directly related to somitogenesis. For example, the process of diffusion, in which molecules move passively from a region of high concentration to low concentration, has been studied in the context of generic negative feedback loops [46]–[48]. The other main mechanism of intracellular molecular movement is active transport, in which molecules move along cytoskeletal elements, typically from where concentration is low to where it is high, a process requiring energy and mediated by motor proteins such as kinesins or dyneins [49], [50]. The impact of active transport on the spatial distribution of intracellular molecules has so far been little explored [51], [52]. Given that chemical reaction systems, including transcriptional control systems, are subject to stochastic fluctuations, which become particularly significant when the numbers of molecules of the interacting species are small, there has been a growing tendency to incorporate stochastic effects into models of intracellular processes [1], [53]–[57].

In view of the observations in the last paragraph, we adopt, as our purpose in this paper, the derivation and exploration of a mathematical model of the segmentation clock in which the nuclear and cytoplasmic diffusion of molecules is considered explicitly. Our model will focus on neighbouring cells in the zebrafish PSM. We will observe that self-repressing proteins within each cell can oscillate in their concentrations and that the oscillations in neighbouring cells can be synchronised by the positive feedback regulation of Notch signalling. We will demonstrate that these observations hold across a range of values for our model parameters, including diffusion coefficients, and for a variety of cell and nuclear shapes and sizes. For simplicity, we will not incorporate active transport or stochasticity into our model but this will be done in future work.

The format of this paper is as follows. In section 2.1, we describe a biological mechanism that has been proposed for the segmentation clock in zebrafish PSM cells. This mechanism involves a core oscillator component in each cell as well as a signalling component between neighbouring cells. In section 2.2 we translate the core oscillator component into a spatial mathematical model. We simulate this model in section 2.2.1, finding ranges of parameter values that yield oscillatory dynamics. We also present spatial profiles of protein and mRNA, discuss the interaction of the species in our model, and observe that oscillations are robust to variety in cell and nuclear shape and size. In section 2.3 we extend our core oscillator model to include Notch signalling between two neighbouring cells. We simulate this signalling model in section 2.3.1, finding parameter values that enable the oscillations in each cell to synchronise. Further spatial profiles are presented and discussed, and further observations are made on the robustness of our results to variety in cell geometries. Ranges of parameter values that yield synchronised oscillations are given in section 2.3.2. We draw conclusions and consider ways to extend our work in the Discussion.

## Results

### 2.1 The segmentation clock in zebrafish

Somitogenesis is not completely understood in any species but it would appear to be more clearly understood in zebrafish rather than, for example, chicks and mice. Consequently a significant proportion of papers on the mathematical modelling of somitogenesis have focused on zebrafish to date. In keeping with this pattern, we shall restrict our own attention to zebrafish, and in particular to the segmentation clock in the presomitic mesoderm (PSM) of zebrafish. In future work, we will expand this perspective by considering the determination wavefront in zebrafish and by considering somitogenesis in other species.

Experimental evidence enabled Lewis to propose a simple control mechanism for the segmentation clock in zebrafish [1]. Correct somite formation is dependent on oscillatory gene expression in individual PSM cells but oscillations between adjacent cells must seemingly also be synchronised. Oscillations within a cell can be controlled by genes subject to a negative feedback loop, where the gene products inhibit transcription by binding to their promoters, whilst Notch signalling between adjacent cells can synchronise oscillations in these cells. The activity of genes that are not part of this control mechanism can be regulated if, for example, they are downstream of the Notch signalling pathway.

The specifics of the proposed mechanism of Lewis are as follows. Two proteins, Her1 and Her7, combinatorially inhibit their own genes as well as the expression of the *deltaC* gene. Meanwhile, the DeltaC protein is exported to the cell membrane where it binds to the Notch receptor on a neighbouring cell. The Notch-DeltaC complex is then cleaved in two separate locations. An intracellular fragment of Notch (called Notch intracellular domain or NICD) in the neighbouring cell breaks off and translocates to the nucleus where it positively regulates the expression of the *her1* and *her7* genes.

By taking into account time delays for transcription and translation, Lewis created a delay differential equation (DDE) model for his proposed mechanism [1]. By exploring this model numerically and analytically, he found that the negative feedback of the inhibitory behaviour of Her1 and Her7 results in oscillatory expression of *her1*, *her7*, and *deltaC* within a cell, whilst the positive feedback of Notch-DeltaC signalling synchronises the oscillations in neighbouring cells. Subsequent experimental work by Lewis and co-workers has provided corroborative evidence for the proposed mechanism and has allowed it to be refined [4], [6].

We shall adopt the proposed mechanism of Lewis in this paper. However, our investigation of the mechanism will be new, for as we mentioned in the Introduction, we shall consider the explicit diffusion of molecules within cells. Unlike Lewis, we shall also model Notch and NICD explicitly.

### 2.2 The core oscillator

**Figure 2:**
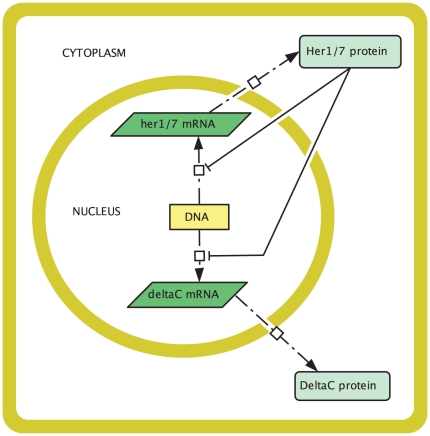
Cell circuitry schematic for the zebrafish segmentation clock core oscillator mechanism. In the nucleus, the *her1*, *her7*, and *deltaC* genes are transcribed to produce *her1*, *her7*, and *deltaC* mRNA respectively. These mRNAs diffuse into the cytoplasm where they are translated to produce Her1, Her7, and DeltaC proteins. The Her1 and Her7 proteins then diffuse into the nucleus and combinatorially inhibit the transcription of the *her1*, *her7*, and *deltaC* mRNAs. The DeltaC protein diffuses to the cell membrane and does not enter the nucleus. Zebrafish PSM cells vary in shape. For simplicity we chose a square cell for this schematic.

Let us begin by considering a mathematical model for the core oscillator, that is, for the oscillatory dynamics within an individual PSM cell. A cell circuitry schematic is given in figure 2. This schematic is consistent with the mechanism of Lewis, as decribed in the previous section (but see also figure 1B in [1] and figure 1B in [5]). There are, additionally, some spatial assumptions. Proteins are translated from mRNA by ribosomes in the cytoplasm. This process is likely to occur at least some minimal distance from the nucleus, so we assume that Her1, Her7, and DeltaC are translated some minimal distance 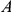 from the nucleus. Since Her1 and Her7 proteins function as transcription factors, we assume that they can diffuse in both the cytoplasm and the nucleus. By contrast, DeltaC is synthesised for export to the cell membrane, so it is unlikely to diffuse into the nucleus. Hence we assume that DeltaC is absent from the nucleus. Finally, unlike the DDE model of Lewis [1], [6], we will not include time delays for transport, transcription, and translation in our model. The duration of these processes will be accounted for in our diffusion, transcription, and translation rates.

It is worth commenting on our assumption, made for simplicity, that molecular movements are modelled only by diffusion. After all, as noted in the Introduction, proteins can also be actively transported from one region in a cell to another. For example, DeltaC proteins are transported to the Golgi body after being synthesised before moving to the cell membrane. Our model assumptions are sufficient to qualitatively capture this behaviour. By assuming that DeltaC proteins cannot enter the nucleus but can only diffuse in the cytoplasm, we ensure that they will move from where their concentration is high (near the nucleus, where they are created) to where their concentration is low (the cell membrane). Similarly our assumptions are sufficient to capture the behaviour of Her1 and Her7 proteins; by assuming that Her1 and Her7 can diffuse in the whole cell and can enter the nucleus, we ensure that at least some of them will enter the nucleus. Active transport of DeltaC to the cell membrane, and of Her1 and Her7 from the cytoplasm into the nucleus, will be considered in future work.

The cell schematic in figure 2 is split into two compartments, the cytoplasm and the nucleus. The equations for our model are different in the different compartments. Our model will consist of a system of partial differential equations (PDEs) with two independent space variables, 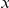 and 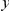. Let 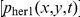 and 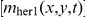 denote, respectively, the concentrations at time 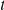 of the Her1 protein and *her1* mRNA at the point 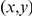 in the cell. For ease of notation, denote 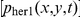 as 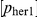 and 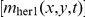 as 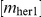.

In the cytoplasm, suppose that Her1 diffuses with diffusion coefficient 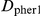 and that it degrades with per molecule degradation rate 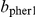. Suppose also that the translation rate per *her1* mRNA molecule is 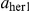. To model our assumption above that Her1, Her7, and DeltaC are synthesised at least some minimal distance 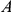 from the nucleus, we define 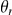 as follows: if 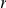 is the distance of a point 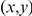 in the cytoplasm from the nucleus, then 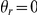 when 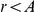 and 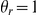 otherwise. By our assumptions we find that 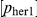 and 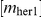 satisfy the following PDE in the cytoplasm: 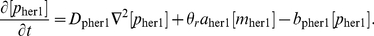
(1)


Now suppose in the cytoplasm that *her1* mRNA diffuses with diffusion coefficient 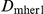 and that it degrades with per molecule degradation rate 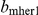. Then 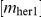 satisfies the following PDE in the cytoplasm: 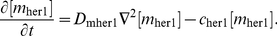
(2)


By replacing “
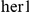
” by “
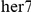
” (respectively, by “
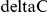
”) in (1) and (2), we obtain PDEs for the Her7 protein and *her7* mRNA (respectively, for the DeltaC protein and *deltaC* mRNA) in the cytoplasm.

In the nucleus, we suppose that the Her1 and Her7 proteins are governed by the same PDEs as in the cytoplasm, with the omission of their terms for translation (translation does not occur in the nucleus). We also assume (as mentioned above) that the DeltaC protein is absent from the nucleus since it is created for export to the cell membrane. We assume that the PDE governing *her1* mRNA (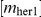) in the nucleus is the same as in the cytoplasm (equation (2)), with the addition of a transcription term in the right hand side of (2) that incorporates combinatorial inhibition due to the Her1 and Her7 proteins. This transcription term is: 
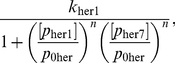
(3)where 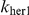 is the basal transcription rate of *her1* mRNA or the transcription rate in the absence of inhibition from the Her1 and Her7 proteins, 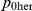 is the concentration of Her1 and Her7 that reduces the transcription rate to half its basal value 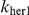, and 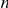 is a Hill coefficient that determines the strength of the inhibition. A larger Hill coefficient implies greater nonlinearity, or co-operativity, in the interaction between the inhibitory protein and the promoter for the gene being inhibited [45]. The transcription rates for *her7* mRNA and *deltaC* mRNA are also assumed to obey (3), except that the basal transcription rate for *her1* mRNA (namely 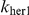) is replaced respectively by basal transcription rates for *her7* and *deltaC* mRNA (namely 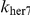 and 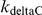). We suppose in the nucleus that *her7* mRNA and *deltaC* mRNA satisfy the same PDEs as they do in the cytoplasm but with the addition of their respective transcription terms in their right hand sides.

Notice that we have assumed that *her1*, *her7*, and *deltaC* mRNAs can be produced at any point in the nucleus. We make this assumption for simplicity because, in fact, zebrafish have only two genes each for *her1*, *her7*, and *deltaC*, so that transcription of each of these mRNAs will only take place at two sites within the nucleus. It is not unusual to make simplifying assumptions of this type; for example, a number of studies have treated the nucleus as a well-mixed compartment [46]–[48]. However, we will, in future work, extend our model to incorporate transcription at specific sites in the nucleus.

The full PDE system for our core oscillator model is stated explicitly in the Supporting Information (Text S1) available online with this article. In order to have a well-defined model, we require initial conditions and boundary conditions. As in the DDE model of Lewis [1], [6], we choose zero initial conditions, that is, we choose all initial mRNA and protein concentrations in both the nucleus and cytoplasm to be zero. In terms of boundary conditions, we choose zero flux at the cell membrane, ensuring that no molecules are exported across it. At the nuclear membrane we choose zero flux for the DeltaC protein to prevent it from entering the nucleus. For all other variables, we choose continuity of flux at the nuclear membrane, allowing import into and export out of the nucleus. The boundary conditions are stated more explicitly in the Supporting Information (Text S1).

#### 2.2.1 Core oscillator simulations

Our core oscillator model can be solved numerically by the finite element method as implemented by the computer package COMSOL (see the section on Experimental Procedures below).

Estimating the model parameters would seem to be a prerequisite for performing simulations. However, our model is new and not all of the parameters have been measured experimentally. For example, the diffusion coefficients have not been measured. Fortunately we can overcome the difficulty of parameter estimation by a process called non-dimensionalisation [58]. This process first involves rescaling the variables in our model by positive constants called reference values. For example, time 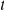 can be rescaled by dividing it by a reference time 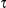, and the spatial variables, 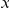 and 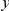, can be rescaled by division by a reference length 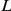. The rescaled variables have no units associated with them. Trivial calculations allow us to rewrite our model in terms of the rescaled variables and in terms of parameters that have no unit or dimension associated with them. This rewritten model is said to be non-dimensionalised; the non-dimensionalised model is qualitatively identical to the original dimensional model.

Next we can choose parameter values such that the non-dimensionalised model, solved on a cell in which distance is defined in terms of the non-dimensionalised spatial variables, yields dynamics in qualitative agreement with the oscillatory dynamics in an individual zebrafish PSM cell. We can then choose the reference length 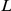 to be such that our simulated cell has the size of a zebrafish PSM cell when distance is written in terms of the dimensional spatial variables. We can also choose the reference time 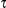 to be such that the period of oscillations in our simulations is equal to the experimentally observed oscillatory period of 30 minutes at 28°C [1] when written in terms of the dimensional time variable. (Notice that the period is known to depend on the temperature at which the zebrafish embryos develop - a warmer temperature results in a shorter period [59].)

Using our reference length and time, as well as our parameter values for the non-dimensionalised model and biological measurements that are available, we can calculate the remaining reference values and values for the parameters in the dimensional model. Then we will have found parameter values in the dimensional model such that this model will yield oscillatory dynamics with the experimentally observed period in a cell the size of a zebrafish PSM cell.

We have non-dimensionalised our core oscillator model, discovered non-dimensional parameters that yield sustained oscillations, and calculated values for the dimensional parameters in the Supporting Information. Parameter values that yield sustained oscillations are the exception, not the rule.

**Figure 3:**
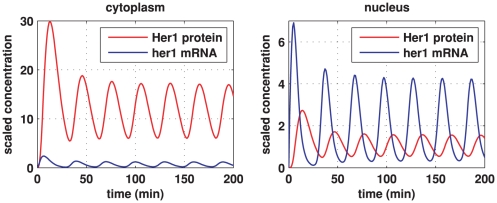
Core oscillator model simulation results showing sustained oscillations. Here we plot Her1 protein (red) and *her1* mRNA (blue) concentrations over time. The left plot shows concentrations in the cytoplasm and the right plot shows concentrations in the nucleus. The concentrations are scaled by reference values. Thus, multiplying the Her1 protein concentration by 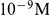 and the *her1* mRNA concentration by 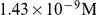 gives the true concentrations. All parameter values are stated in the Supporting Information. The other species in the core oscillator model show qualitatively similar behaviour.

**Figure 4:**
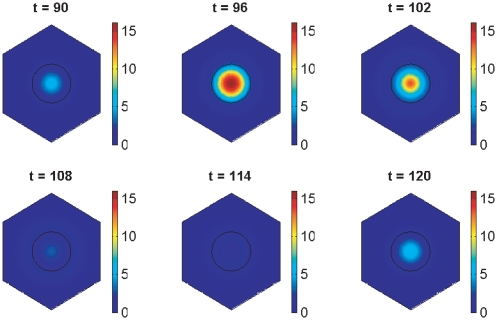
Core oscillator model simulation results showing spatial distributions of *her1* mRNA. The plots show *her1* mRNA concentration at six minute intervals over the fourth period of oscillations by which time transient behaviour has died down. The concentration is scaled by a reference value (multiplying the concentration by 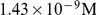 gives the true concentration). Parameter values are stated in the Supporting Information.

**Figure 5:**
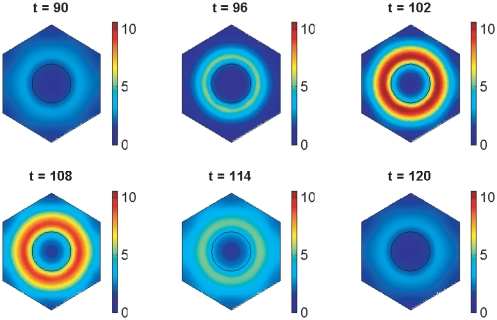
Core oscillator model simulation results showing spatial distributions of Her1 protein. The plots show Her1 protein concentration at six minute intervals over the fourth period of oscillations by which time transient behaviour has died down. The concentration is scaled by a reference value (multiplying the concentration by 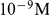 gives the true concentration). Parameter values are stated in the Supporting Information.

Simulations for our non-dimensionalised core oscillator model, performed on a hexagonal cell with a circular nucleus, are demonstrated in figures 3, 4, and 5, and also in two Supporting Information files (see “Animation S1” and “Animation S2”). Figure 3 demonstrates stable oscillations over time in the concentrations of Her1 protein and *her1* mRNA both in the cytoplasm and the nucleus. Notice that the concentration of Her1 protein is higher in the cytoplasm than the nucleus. We will be interested to see if this balance holds when we consider the active transport of Her1 (and Her7) into the nucleus in future work.

As we noted in the Introduction, transcriptional control systems, such as the zebrafish segmentation clock, are subject to stochastic effects or noise arising from random fluctuations in the regulation of gene expression [53], [60]. The binding and dissociation of a gene regulatory protein (such as Her1 or Her7) to and from its site on DNA are stochastic processes, each described by a certain probability of occurrence per unit time [1]. For large numbers of proteins, these processes can be approximated by deterministic mathematical models, such as those considered in this paper. However, for small numbers of proteins, this approximation becomes less accurate. To gain insight into the validity of our approach, it is therefore sensible to calculate how many Her1 and Her7 molecules are typically present in the nucleus in our simulations. From the right plot of figure 3, we see that the average concentration of Her1 protein in the nucleus is about 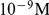. But as mentioned above, a zebrafish PSM cell has a nuclear diameter of roughly 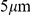, corresponding to a volume of roughly 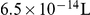 if the nucleus is spherical. By a standard calculation, we then find that the average number of Her1 protein molecules in the nucleus is about 40. Similarly the average number of Her7 protein molecules in the nucleus is around 40. These numbers are not low enough to invalidate our modelling approach. Yet they are sufficiently low for us to consider incorporating stochastic effects into our model in future work. Lewis has considered the role of noise in his DDE model of the zebrafish segmentation clock [1].

Figures 4 and 5 show how the concentrations of *her1* mRNA and Her1 protein vary spatially throughout the cell over the fourth period of the oscillations in figure 3 when the model has converged to stable oscillatory behaviour. At the start of this period (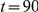 minutes), the concentration of *her1* mRNA is low. However, the concentrations of Her1 and Her7 proteins are also low, so that 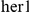 mRNA is little affected by negative feedback at this time. Consequently, 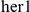 mRNA levels rise, leading to increased production of Her1 protein in the cytoplasm (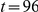 minutes). Similarly, Her7 protein levels rise in the cytoplasm. Her1 and Her7 proteins then migrate into the nucleus and inhibit transcription (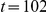 minutes). With transcription inhibited, mRNA synthesis is reduced, as a result of which there is less mRNA to create protein, so protein levels fall (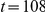 minutes). As protein levels fall, protein inhibition of transcription lessens, so transcription rates rise causing more mRNA to be made (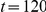 minutes). This pattern now repeats. Our results for Her7 and DeltaC proteins are similar to those for Her1 (except that DeltaC is absent from the nucleus by assumption) and are hence omitted.

We performed simulations on a hexagonal cell because zebrafish PSM cells are often this shape, though they can also be approximately rectangular or rounded [61]. For our parameter choices, the oscillatory dynamics that we have obtained are robust to changes in the cell and nuclear shapes - oscillations can occur for circular, elliptical, square, and hexagonal cell shapes, as well as for circular and elliptical nuclei (see the Supporting Information). Oscillations are also robust to changes in the size of the nucleus or the cell. However, we have noticed that when the nuclear size is decreased, then the average mRNA and protein concentrations are also decreased. In any case our model predicts robustness of oscillations to changes in the shape or size of the cell or nucleus. This is a prediction that should be expected, given that our model is based on experimental evidence that zebrafish PSM cells exhibit oscillatory dynamics despite the fact that these cells must inevitably vary a little in shape and size. Our observations on cell geometry are only possible because of our explicitly spatial method of modelling. Biological pattern formation would be impossible without a certain robustness to cell shape.

The values that we have found for the dimensional parameters are: all diffusion coefficients equal 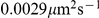, all degradation rates equal 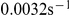, all translation rates equal 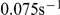, all basal transcription rates equal 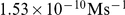, the concentration of Her1 and Her7 that reduces the transcription rate to half its basal value is 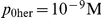, the minimum distance of translation from the nucleus is 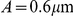, and the Hill coefficient 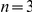. These dimensional values were found using the assumptions that a zebrafish PSM cell has a length of roughly 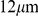 and a nucleus of diameter 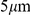, assumptions consistent with the literature and recent experimental evidence [1], [31], [61], [62]. To obtain our dimensional parameter values, we used biological estimates in [1], [6] for rates such as transcription and translation. Given that the different mRNA and protein species in the core oscillator all seem to be controlled by the same negative feedback loop, it is not necessarily inappropriate to suggest, for example, that they have the same diffusion coefficient or the same degradation rate. Moreover, Lewis simulated his DDE model by assuming that all degradation rates were identical in [1] or almost identical in work with Ozbudak in [6].

To our knowledge, diffusion coefficients for the species involved in zebrafish somitogenesis are yet to be measured experimentally. We therefore hope that our estimates inspire others to measure these coefficients. Measurements of diffusion coefficients of proteins within cytoplasm, nucleus, or within membranes suggest that our estimates are of the right order of magnitude, particularly given that our estimates (except for DeltaC protein) incorporate diffusion through the nuclear membrane [63]–[65]. In future work, we will model transport across the nuclear membrane explicitly by taking the structure of the membrane into account.

Although Lewis uses a Hill coefficient of 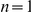 in his DDE model [1], [6], we were unable to find sustained oscillatory dynamics for 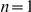. We have found sustained oscillatory dynamics for 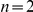 (with different values for the other model parameters than those stated above) but oscillations appear to be less robust to changes in the parameters for 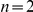 than for 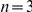. A Hill coefficient of 3 implies there is some co-operativity in the interaction between the inhibitory protein and the promoter for the gene being inhibited [45]. For simplicity we have investigated only integer Hill coefficients. Discussion of the meaning and use of non-integer Hill coefficients may be found in [66], [67]. The interactions governing somitogenesis in mice appear to be more complicated than in zebrafish. Nevertheless it is of interest to note that the Hill coefficient for the Hes1 oscillator in mouse somitogenesis has been estimated by Zeiser et al to be about 3 [66].

For a given binding process between two species, there is an association between the number of binding sites and the Hill coefficient quantifying their interactions [39], [41]. Hence studies of the number of sites where Her proteins bind to their gene promoters in zebrafish [68] are of potential interest in understanding the role of the Hill coefficient in our model. However, the precise nature of the relationship between binding sites and Hill coefficients remains a matter for discussion [69].

Finally we have explored ranges of parameter values for which oscillations persist to 900 minutes when the cell is hexagonal as in figures 4 and 5. By assuming that all the diffusion coefficients are equal and holding all the other parameter values fixed, we have found that sustained oscillations occur for diffusion coefficients in the range 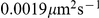 to 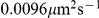, or 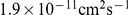 to 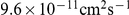. By assuming all the degradation rates are equal and holding all the other parameter values fixed as above, we have found that sustained oscillations occur for degradation rates in the range 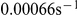 to 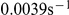. Varying the Hill coefficient 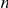 but keeping the other parameters fixed as above, we find sustained oscillations for 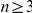. We have found ranges of values for the other model parameters such that sustained oscillations occur; these ranges are given in table 1. See section 2.3 in the Supporting Information file “Text S1” for more details on our method of numerical exploration.

**Table 1:** Parameter ranges giving sustained oscillations in the core oscillator model.

Parameter	Range giving sustained oscillations
Diffusion coefficients (all species)	0.0019 to 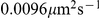
Degradation rates (all species)	0.00066 to 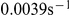
Translation rates (of Her1, Her7, DeltaC proteins)	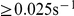
Basal transcription rates (of *her1*, *her7*, *deltaC* mRNAs)	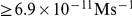
Minimal distance 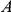 of translation from nucleus	0 to 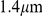
Critical concentration 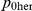 of Her1 and Her7 proteins	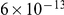 to 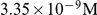
Hill coefficient 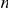	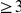

Ranges of parameter values in the core oscillator model that yield sustained oscillatory dynamics (simulating on a hexagonal cell up to 900 minutes). The ranges here were calculated from table 1 in the Supporting Information file “Text S1”, as explained in section 2.3 in that file.

### 2.3 Notch signalling

**Figure 6:**
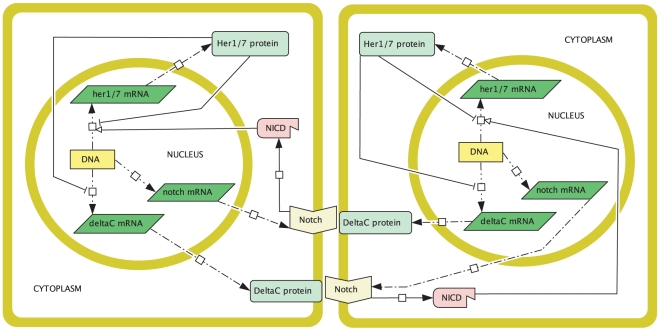
Cell circuitry schematic for Notch signalling between two neighbouring zebrafish PSM cells. Inside each cell, the core oscillator mechanism described in figure 2 holds with the exception that Notch signalling positively regulates the expression of the *her1* and *her7* genes. The signalling mechanism is as follows. In the nucleus, *notch* mRNA is transcribed, which then diffuses into the cytoplasm and produces Notch proteins by translation. These Notch molecules diffuse to the cell membrane where they function as receptors. DeltaC proteins from one cell bind to Notch receptors in the neighbouring cell. Each Notch-DeltaC complex is cleaved in two separate locations, causing an intracellular fragment of Notch (called Notch intracellular domain or NICD) to break off and translocate to the nucleus where it upregulates *her1* and *her7* gene expression. Zebrafish PSM cells vary in shape. For simplicity we chose square cells for this schematic.

In section 2.2 we discussed a model for the core oscillator mechanism in an individual zebrafish PSM cell. In this section we extend the core oscillator model to construct a model for two neighbouring PSM cells which communicate by Notch signalling. Although the role of Notch signalling in zebrafish somitogenesis is not completely understood, a mechanism that fits experimental observations has been proposed by Lewis to explain it [1], [6]. We outlined this mechanism in section 2.1. Figure 6 shows a cell circuitry schematic consistent with the proposed mechanism. We shall use figure 6 to construct our model for two neighbouring PSM cells. Note that we shall attach the subscript 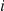 to dependent variables in cell 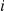 (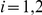) to show that these variables are appropriate to that cell.

We begin our description of the signalling model by noting (as in figure 6) that, inside each cell, the core oscillator mechanism holds with the exception that the signalling upregulates the expression of the *her1* and *her7* genes. Thus, the nuclear and cytoplasmic PDEs of the core oscillator model hold in each cell except that the transcriptional terms for the *her1* and *her7* mRNA PDEs in the nucleus are adjusted.

To be more specific, the signalling causes Notch intracellular domain (NICD) to be released into each cell, as explained in figure 6. If we let 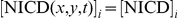 be the concentration of NICD at the point 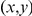 in cell 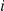 (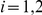) at time 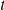, then the transcriptional term for *her1* mRNA in (3) becomes the following in cell 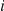: 
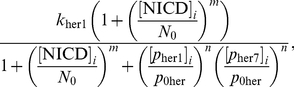
(4)where 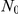 is a threshold concentration of NICD above which its impact on transcription is stronger and where 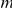 is a Hill coefficient. The new transcriptional term is an increasing function of NICD; the more signalling, the more NICD will be released into each cell, and the greater the upregulation of *her1* expression. On the other hand, there are physical limitations on how quickly transcription can occur, which is reflected in the transcriptional term in (4) by allowing it to saturate at the value 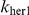 as the NICD concentration becomes suitably large. Ozbudak and Lewis consider a transcriptional term as in (4) (with 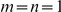) in a recent DDE model [6], updated from a slightly different transcriptional term in an earlier DDE model of Lewis [1]. Our transcriptional term for *her7* mRNA in the presence of signalling is identical to (4) except that the basal transcription rate 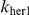 is replaced by 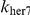. We asume, as in [6], that the transcriptional term for 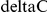 mRNA is unchanged in the presence of signalling.

Now let us account for the creation and diffusion of the signalling. The signalling mechanism is outlined in figure 6. In the nucleus in each cell, we assume that *notch* mRNA is transcribed at a rate 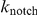, that it diffuses at a rate 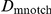, and that it degrades at a rate 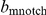. The diffusion of *notch* mRNA causes it to move out from the nucleus into the cytoplasm, where it continues to diffuse and degrade. In the cytoplasm, *notch* mRNA produces Notch protein by translation at a rate 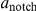. For Notch translation, we retain the assumption from the core oscillator model that translation occurs some minimal distance 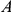 from the nucleus. In the cytoplasm, Notch proteins diffuse at a rate 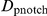 and degrade at a rate 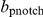. The Notch proteins are created to serve as receptors at the cell membrane, so we assume that they cannot diffuse into the nucleus. It is trivial to convert the assumptions in this paragraph into PDEs (see the Supporting Information).

A little biological discussion is needed before we can make further mathematical comments. Once Notch proteins reach the cell membrane they function as receptors by binding DeltaC proteins displayed at the surface of a neighbouring cell. After binding, Notch releases its internal fragment NICD, which translocates to the nucleus and initiates the transcriptional response described above, and DeltaC protein (attached to an extracellular fragment of Notch called Notch extracellular domain or NECD) is endocytosed in the cell where it was synthesised, an act believed necessary for the release of NICD [70]. It has been suggested that DeltaC is recycled after endocytosis [71], but since this has not been conclusively demonstrated yet we shall, for simplicity, assume that endocytosed DeltaC plays no role in our signalling model. Hence for modelling purposes we shall assume that, at the cell membrane of each cell, DeltaC is lost at the rate at which it binds to Notch.

A Notch receptor is irretrievably altered by binding its ligand, so it is believed that each receptor can fire only once after it has bound its ligand [72]. Hence we assume that, at the cell membrane of each cell, Notch is lost at the rate at which it binds to DeltaC and NICD is released at this same rate.

We model the communicating cell membranes in figure 6 as a narrow region or strip, which we call the membrane subdomain. Although molecules do not tend to wander freely between the cell membranes of neighbouring cells, it is not unreasonable, in terms of spatial modelling, to treat the communicating cell membranes as a single region between which molecules can wander freely, provided this region is sufficiently narrow and only realistic molecular interactions are permitted. We suppose that DeltaC and Notch from each cell diffuse into the membrane subdomain (with diffusion coefficients 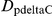 and 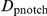 respectively) where they then continue to diffuse (proteins are known to roam within a cell membrane) and where they are subject to natural degradation (at rates 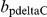 and 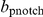 respectively).

It is reasonable to assume in the membrane subdomain that the rate of binding between cell 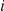 (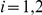) Notch receptors and cell 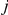 (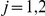, 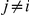) DeltaC proteins is low if the concentration of cell 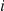 Notch receptors is low, and is proportional (with constant of proportionality 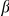, say) to the concentration of cell 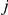 DeltaC proteins if the concentration of cell 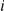 Notch receptors is high. Then, if we let 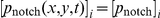 denote the concentration of cell 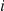 Notch at the point 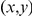 at time 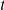 and let 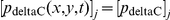 denote the concentration of cell 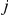 DeltaC at the point 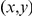 at time 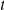, the following choice for the binding rate presents itself: 
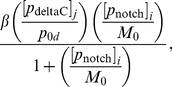
(5)where 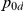 and 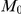 are threshold concentrations with intuitive interpretations. In keeping with the biological discussion above, we assume in the membrane subdomain that cell 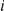 Notch and cell 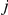 DeltaC are both lost at the binding rate (5) and that cell 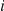 NICD is released at the binding rate (5). Although it does not influence our assumptions, it is of interest to note that cell-surface levels of Notch protein are believed to be generally much higher than levels of DeltaC protein. On the timescale of the segmentation clock, cell-surface levels of Notch are unlikely to change significantly, an idea that is implicitly assumed in the DDE model of Lewis [1], [6].

To complete our description of the signalling mechanism, we must explain how cell 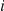 NICD translocates from the membrane subdomain to the nucleus of cell 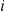. Thus, we assume in the membrane subdomain that cell 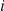 NICD diffuses with diffusion coefficient 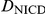 and degrades with degradation rate 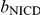. Cell 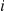 NICD is allowed to diffuse into the cytoplasm of cell 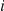 (but not cell 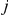), where it continues to diffuse and degrade, eventually reaching the nucleus where it upregulates *her1* and *her7* transcription as in (4). NICD is not used up by its transcriptional role; however, it does continue to diffuse and degrade within the nucleus.

The full set of PDEs for our signalling model is stated in the Supporting Information. For ease of reference, and given that a central and new feature of our modelling approach is the explicit interaction and movement of molecules at the cell membrane, we also state here the PDEs for DeltaC, Notch, and NICD in the membrane subdomain:
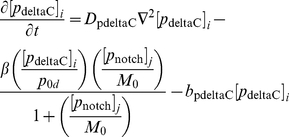
(6)

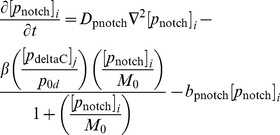
(7)

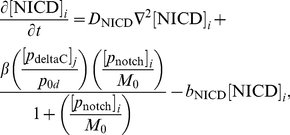
(8)where 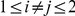.

As we did for the core oscillator model in section 2.2, we suppose, for the signalling model, that the initial concentration of all species is zero. Our boundary conditions for the signalling model are chosen to be consistent with the above description of this model; they are stated in the Supporting Information.

#### 2.3.1 Notch simulations

We explore the Notch signalling model in a manner analogous to the exploration of the core oscillator model in section 2.2.1. Thus we begin by observing that the Notch signalling model can be solved numerically using COMSOL. Not all of the model parameters have been measured experimentally, so parameter estimation is not easy, but we can circumvent this issue by simulating a non-dimensionalised version of our model, seeking non-dimensional parameters that produce numerical results that fit qualitatively with experimental observations. Computationally it is straightforward to control the time when transcription begins in each cell and when signalling begins between the two cells. We seek non-dimensional parameters such that each cell exhibits oscillatory dynamics in the absence of signalling but such that oscillations in the two cells are forced to synchronise after signalling begins.

Since each cell in the signalling model contains a core oscillator mechanism, we retain the non-dimensional parameter choices made in simulating the core oscillator model, which allows us to retain the oscillatory dynamics discussed in section 2.2.1. Numerical investigation allows us to find values for the remaining parameters such that the oscillations in the two cells synchronise. Parameter values that cause oscillations to synchronise are the exception, not the rule. As we did for the core oscillator model, we can use non-dimensional parameter values, reference values for length and time, and available biological measurements to calculate values for the dimensional parameters in the signalling model. All of this has been done in the Supporting Information.

**Figure 7:**
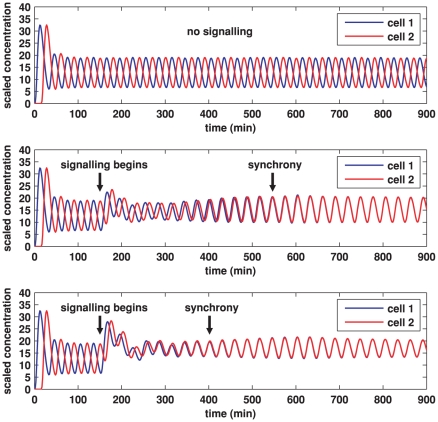
Plots of Her1 protein concentration produced from simulations of the Notch signalling model. Each plot shows the total concentration of Her1 protein in cell 1 (blue) and cell 2 (red) over time. The concentration is scaled by a reference value (multiplying the concentration by 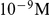 gives the true concentration). In each plot, transcription begins in cell 1 at time 0 and in cell 2 when half the core oscillator period (15 minutes) has elapsed. All parameter values are stated in the Supporting Information. **Top**: no signalling. The cells oscillate perfectly out of synchrony. **Middle**: Notch signalling begins at time 150 minutes. Oscillations in the two cells synchronise by 550 minutes and the average concentration is increased by approximately 20% compared to the case (top plot) in which no signalling occurs. **Bottom**: Notch signalling begins at time 150 minutes but here we reduce by a factor of 10 (relative to the middle plot) the threshold concentration 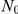 of NICD above which its impact on transcription is stronger. The oscillations synchronise by 400 minutes, the average concentration is further increased, and the amplitude of the oscillations is notably reduced.

**Figure 8:**
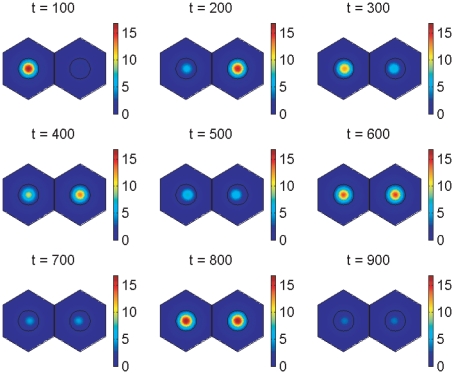
Notch signalling model simulation results showing spatial distributions of *her1* mRNA. The plots show *her1* mRNA concentration at 100 minute intervals. The concentration is scaled by a reference value (multiplying the concentration by 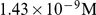 gives the true concentration). The parameters used to create the middle plot in figure 7 are used here (see Supporting Information). Signalling between the cells begins at 150 minutes and has clearly synchronised their behaviour by 600 minutes.

**Figure 9:**
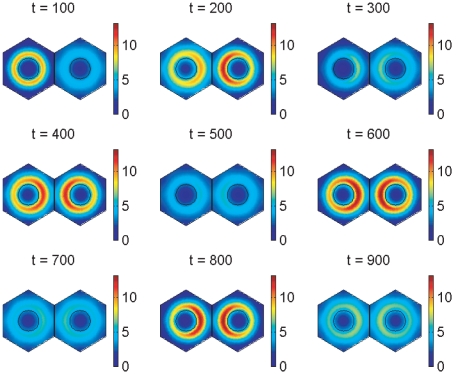
Notch signalling model simulation results showing spatial distributions of Her1 protein. The plots show Her1 protein concentration at 100 minute intervals. The concentration is scaled by a reference value (multiplying the concentration by 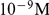 gives the true concentration). The parameters used to create the middle plot in figure 7 are used here (see Supporting Information). Signalling between the cells begins at 150 minutes and has clearly synchronised their behaviour by 600 minutes.

**Figure 10:**
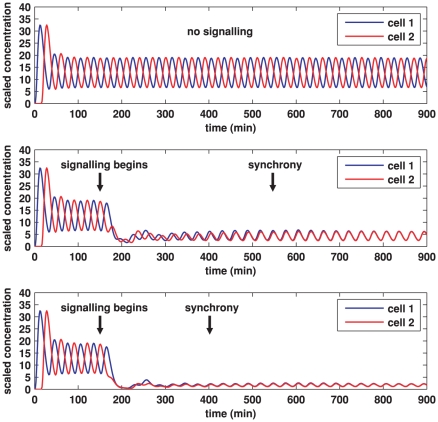
Plots of DeltaC protein concentration produced from simulations of the Notch signalling model. Each plot shows the total concentration of DeltaC protein in cell 1 (blue) and cell 2 (red) over time. The concentration is scaled by a reference value (multiplying the concentration by 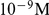 gives the true concentration). In each plot, transcription begins in cell 1 at time 0 and in cell 2 when half the core oscillator period (15 minutes) has elapsed. All parameter values are stated in the Supporting Information. **Top**: no signalling. The cells oscillate perfectly out of synchrony. **Middle**: Notch signalling begins at time 150 minutes. Oscillations in the two cells synchronise by 550 minutes and, compared to the case (top plot) in which no signalling occurs, the average concentration is decreased by a factor of 3 and the amplitude of the oscillations is notably reduced. **Bottom**: Notch signalling begins at time 150 minutes but here we reduce by a factor of 10 (relative to the middle plot) the threshold concentration 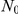 of NICD above which its impact on transcription is stronger. The oscillations synchronise by 400 minutes, the average concentration is further decreased, and the amplitude of the oscillations is further reduced.

**Figure 11:**
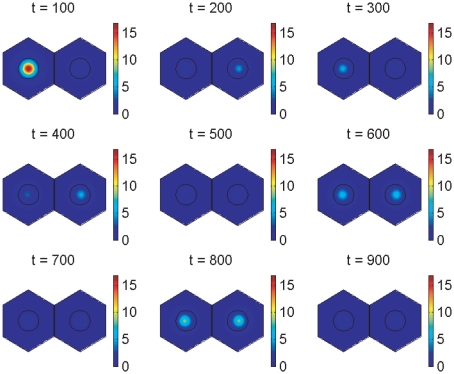
Notch signalling model simulation results showing spatial distributions of *deltaC* mRNA. The plots show *deltaC* mRNA concentration at 100 minute intervals. The concentration is scaled by a reference value (multiplying the concentration by 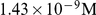 gives the true concentration). The parameters used to create the middle plot in figures 7 and 10 are used here (see Supporting Information). Signalling between the cells begins at 150 minutes and has synchronised their behaviour by 600 minutes.

**Figure 12:**
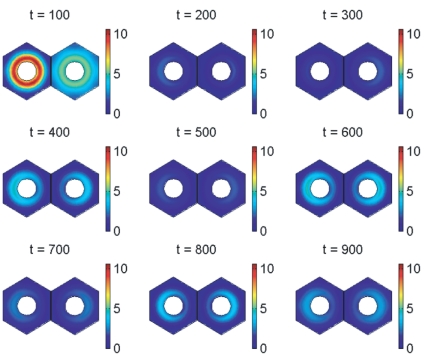
Notch signalling model simulation results showing spatial distributions of DeltaC protein. The plots show DeltaC protein concentration at 100 minute intervals. The concentration is scaled by a reference value (multiplying the concentration by 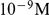 gives the true concentration). The protein is absent from the nucleus by our modelling assumptions. The parameters used to create the middle plot in figures 7 and 10 are used here (see Supporting Information). Signalling between the cells begins at 150 minutes and has synchronised their behaviour by 600 minutes.

Simulations for our signalling model, performed on hexagonal cells with circular nuclei, are demonstrated in figures 7, 8, 9, 10, 11, and 12, and also in two Supporting Information files (see “Animation S3” and “Animation S4”). We denote as cells 1 and 2 the cells that are on the left and right respectively in figures 8, 9, 11, and 12.

Figure 7 demonstrates the total cell concentration of Her1 protein in cells 1 and 2 over time in three cases. In all cases, transcription begins in cell 1 at time 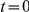 and in cell 2 when half the core oscillator period (15 minutes) has elapsed. In the top plot, signalling is not allowed for the entire simulation. Since the core oscillators in each cell are identical, the oscillations in the two cells remain perfectly out of synchrony. In reality no two cells are identical, so that we might expect different cells to have slightly different oscillatory periods and to slowly drift in and out of synchrony in the absence of signalling. This would not necessarily prevent Notch signalling from synchronising oscillations in neighbouring cells, a result established in the DDE model of Lewis [1] and confirmed in our PDE model in the Supporting Information.

In the middle plot in figure 7, signalling begins at time 150 minutes, with synchrony occurring by 550 minutes. The oscillatory period is slightly (but not significantly) reduced by Notch signalling. This numerical result is consistent with recent experimental observations, based on time-lapse microscopy, that disruption of Notch-Delta increases the period [73]. Notice that the signalling increases the average concentration of Her1 by approximately 20%. This result is consistent with recent experiments by Ozbudak and Lewis that have suggested that loss of Notch signalling in zebrafish embryos causes a reduction of around 20% in mean Her1 protein levels [6].

Synchrony can occur without significantly raising mean protein concentrations but it takes longer. Synchrony and mean protein and mRNA levels are influenced by varying the parameters, an idea we discuss in reference to the parameter 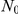 which (recall from section 2.3) is a threshold concentration of NICD above which its impact on transcription is stronger. The impact of decreasing this threshold concentration is shown in the bottom plot of figure 7, which was made in the same way as the middle plot except that 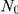 was decreased by a factor of 10. Synchrony occurs faster than in the middle plot and the mean Her1 concentration is increased but the amplitude of the oscillations is reduced. Decreasing 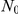 by a factor of 100 relative to the bottom plot of figure 7 causes synchrony to occur faster still (result not shown), increases the mean Her1 concentration further, and forces oscillations to die out altogether - Her1 concentrations stabilise at the same constant value. Loss of oscillations or significantly reduced amplitude in oscillations will disrupt somitogenesis since somite segmentation depends on oscillatory gene expression [4].

Loss of oscillations for small 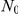 is readily explained in our model. The transcription rate of *her1* mRNA in (4) will saturate at its basal rate 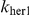 if 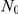 is suitably small, and a similar remark holds for *her7* mRNA. Constant transcription of *her1* and *her7* mRNA will lead to constant translation of Her1 and Her7 proteins. This will prevent oscillatory dynamics in each cell since oscillations in Her1 and Her7 drive the core oscillators.

Mathematically speaking, the impact on transcription of NICD overexpression will be the same as reducing 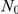, so our comments on the influence of small 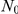 on disrupting somitogenesis apply equally well to the influence of overexpression of NICD. Consistent with these observations is the recent experimental result that heat-shock-triggered overexpression of NICD disrupts the formation of somites [6].

Figures 8 and 9 were created with the same parameter values used to create the middle plot in figure 7 and show respectively how the concentrations of *her1* mRNA and Her1 protein vary spatially throughout the cell at intervals of 100 minutes. Initially the cells oscillate out of synchrony and do not communicate. Notch signalling begins at 150 minutes. Release of NICD from the communicating cell membranes upregulates transcription of *her1* mRNA in the side of each nucleus that is closer to the communicating membranes. Although this is not obvious from figure 8, it is clearly deducible from figure 9 which, at 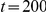 minutes, shows greater concentrations of Her1 protein on the sides of the cells closest to the communicating membranes. This upregulatory influence continues until (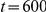 minutes) the two cells are in perfect synchrony.

Figure 10 demonstrates the total cell concentration of DeltaC protein in cells 1 and 2 over time in three cases. These are the same three cases as those presented in figure 7 for Her1 protein. Thus, the top plot in figure 10 shows that DeltaC concentrations oscillate out of synchrony in the two cells when they begin out of synchrony and there is no signalling between them; the middle plot shows that signalling beginning at 150 minutes can synchronise the oscillations in the two cells by 550 minutes; and the bottom plot shows that reducing 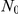, the critical concentration of NICD, hastens the synchrony, which occurs by 400 minutes.

Figure 10 is consistent with figure 7 in that the amplitude of the oscillations is reduced by signalling. However, notice that whilst the average Her1 concentration is increased by signalling (figure 7), the average DeltaC concentration is reduced (figure 10). How do we explain these results? Signalling releases NICD into each cell, which upregulates the transcription of *her1* and 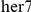 mRNAs by the transcriptional term in (4). This leads to more Her1 and Her7 protein being translated in the cytoplasm. Hence the average concentrations of Her1 and Her7 are increased. The transcription of 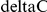 mRNA is governed by negative feedback due to Her1 and Her7 (the transcriptional term for 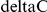 mRNA is (3) where 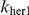 is replaced by 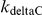). Consequently, increased levels of Her1 and Her7 reduce the average concentration of 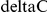 mRNA, which causes less DeltaC protein to be translated in the cytoplasm so that the average concentration of DeltaC is also reduced. DeltaC is further reduced by being lost at the communicating cell membranes when it binds to Notch receptors.

The signalling-induced reduction in 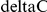 mRNA and DeltaC protein levels is apparent from figures 11 and 12, which show how the concentrations of these species vary spatially throughout the cell at intervals of 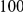 minutes. These figures were created with the same parameter choices used to create the middle plots in figures 7 and 10. Observe in figure 11 that the concentration of 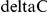 mRNA is lower on the sides of the nuclei closer to the communicating cell membranes. This occurs because its transcription is lower here, due to the dependence of this transcription on negative feedback from Her1 and Her7 and the higher concentration of Her1 and Her7 on the sides of the cells closer to the communicating cell membranes as depicted for Her1 in figure 9. The lower concentration of 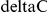 mRNA on the sides of the nuclei closer to the communicating cell membranes causes the translation of DeltaC protein to be lower on these sides of the nuclei, and, as noted in the previous paragraph, DeltaC is lost when it binds to Notch at the communicating cell membranes. These latter observations account for the spatial profiles of DeltaC in figure 12.

Spatial profiles of Notch (results not shown) reveal that its distribution is changed little by signalling. The concentration oscillates at the communicating cell membranes but the changes are scarcely discernible to the naked eye, which is why we do not include these profiles. The amplitude of the oscillations in Notch concentration are small compared to the average concentration in our model, which is consistent with our earlier observation in section 2.3 that, on the timescale of the segmentation clock, cell-surface levels of Notch are unlikely to change significantly.

We discussed the robustness of oscillatory dynamics to changes in the shape and size of the cell and nucleus for our core oscillator model in section 2.2.1. A similar robustness holds in terms of the capacity for Notch signalling to synchronise oscillations in neighbouring cells. Synchronisation can occur for rounded, square, and hexagonal cell shapes, as well as for circular and elliptical nuclei. It can also occur across a range of cell and nuclear sizes. Given that defective pattern formation would probably kill an embryo, it is reassuring that our signalling model possesses a geometrical robustness (see Supporting Information for further discussion and additional figures).

From the non-dimensional parameters used to create figures 8, 9, 11, 12, and the middle plots in figures 7 and 10, we found dimensional parameters. Since our signalling model contains a core oscillator in each cell, the dimensional parameters in our signalling model that relate to these core oscillators are the same as those stated in section 2.2.1. We can summarise all the signalling model dimensional parameters as follows: all diffusion coefficients equal 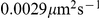, all degradation rates equal 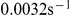, all translation rates equal 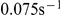, all basal transcription rates equal 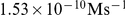, 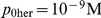, 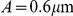, 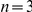, 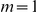, 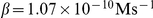, 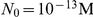, and 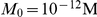.

#### 2.3.2 Parameter ranges that yield synchronised oscillations

In the Notch signalling model, we have explored ranges of parameter values for which oscillations persist and are synchronised by 900 minutes where signalling begins at 150 minutes (simulating on two hexagonal cells as in figure 8). By assuming that all the diffusion coefficients are equal and holding all the other parameter values fixed as above (final paragraph, previous section), we have found that synchronised oscillations occur for diffusion coefficients in the range 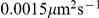 to 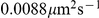, or 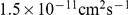 to 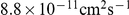. By assuming all the degradation rates are equal and holding all the other parameter values fixed as above, we have found synchronised oscillations for degradation rates in the range 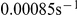 to 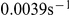. To the extremes of these ranges for diffusion and degradation, the oscillatory amplitude varies and synchrony is not completely perfect.

These ranges for the diffusion and degradation rates are very similar to those needed for sustained oscillations in the core oscillator model (see section 2.2.1 and table 1). However, we obtain a different result for the negative feedback Hill coefficient 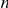. Varying this coefficient whilst keeping the other parameters fixed as above, we find synchronised oscillations by 900 minutes only for 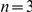 and 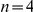. Synchronised oscillations will also occur for 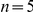 though it takes longer than 900 minutes for exact synchrony to be reached. We have seen that sustained oscillations occur in the core oscillator model for any 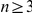 but in the signalling model we have found that if 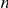 is suitably larger than 3 then signalling has little impact on the dynamics. In fact, for 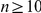, we obtain results very similar to those in the top plot in figure 7 in which no signalling takes place. It would seem that the requirement for oscillations in each core oscillator forces 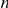 to be bounded below by 3, whilst the requirement for signalling to synchronise these oscillations in a reasonable time bounds 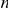 above by 4. There are biological implications: since the Hill coefficient 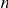 is a measure of co-operativity between the inhibitory proteins (Her1 and Her7) and the genes they inhibit (*her1*, *her7*, *deltaC*), our findings suggest that a certain level of co-operativity is needed for oscillations but that too much co-operativity weakens the impact of Notch signalling. This is a matter that warrants further attention.

Ranges of values for model parameters that yield synchronised oscillations in the Notch signalling model are presented in table 2. Notice how we state in table 2 that there are synchronised oscillations only when the positive feedback Hill coefficient 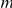 is 1. In fact we have also found that synchronised oscillations occur for 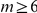. Yet for 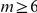 the oscillatory period is tripled by the signalling and the amplitude of the oscillations is increased by a factor of more than 6. Notch signalling is unlikely to have such a dramatic impact on the oscillations, according to experimental studies of the effect of blocking Notch signalling [6]. Hence we have chosen not to include the range 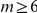 in table 2.

**Table 2:** Parameter ranges giving synchronised oscillations in the Notch signalling model.

Parameter	Range giving synchronised oscillations
Diffusion coefficients (all species)	0.0015 to 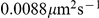
Degradation rates (all species)	0.00085 to 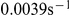
Translation rates (of Her1, Her7, DeltaC, Notch proteins)	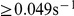
Basal transcription rates (of *her1*, *her7*, *deltaC* mRNAs)	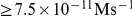
Basal transcription rate 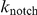 (of *notch* mRNA)	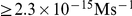
Minimal distance 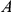 of translation from nucleus	0.24 to 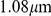
Critical concentration 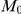 of Notch	0 to 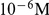
Critical concentration 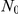 of NICD	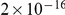 to 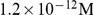
Critical concentration 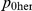 of Her1 and Her7 proteins	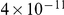 to 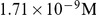
Hill coefficient 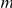	1
Hill coefficient 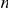	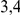

Ranges of parameter values in the Notch signalling model that yield synchronised oscillatory dynamics (simulating on two hexagonal cells up to 900 minutes, where signalling starts after 150 minutes). The ranges here were calculated from table 2 in the Supporting Information file “Text S1”, as explained in section 3.3 in that file.

The idea that signalling can synchronise oscillations whilst dramatically increasing their period and amplitude was encountered by Lewis in exploring his DDE model which our PDE model extends [1]. Lewis explained this result by observing that Notch signalling creates its own feedback loop, which he writes as: activated Notch in cell 1 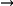 *her1*/*her7* in cell 1 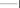 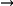 *deltaC* in cell 1 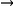 activated Notch in cell 2 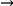 *her1*/*her7* in cell 2 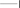 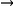 *deltaC* in cell 2 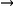 activated Notch in cell 1 (where 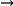 denotes stimulation and 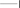 denotes inhibition). For a certain parameter relationship, this long Notch feedback loop can replace, as the driving force behind oscillations, the shorter feedback loop in which Her1 and Her7 inhibit their own synthesis. The DDE model of Lewis uses time delays rather than diffusion to account for transport processes and does not explicitly consider NICD or Notch, so the parameter relationship that corresponds to this switch in driving force is different in the model of Lewis compared to our relationship that 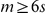. Indeed, the parameter relationship used by Lewis amounts to a qualitative change in the transcriptional control of Her1 and Her7 in which these proteins no longer directly inhibit their own synthesis [1]; a qualitative change of this type does not occur in our model when we increase 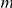 from 1 to be 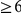. For 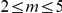, the two cells in our model oscillate together but the concentrations are not synchronised - for example, the levels of Her1 protein are higher in cell 2. There is a biological implication: given that 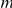 is a Hill coefficient measuring co-operativity in the interaction between NICD and the genes for 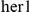 and 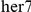, defective somites may form even if this co-operativity is fairly weak (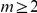).

## Discussion

Mathematical models of somitogenesis have been appearing for over 30 years, evolving in parallel with advances in experimental procedures and discoveries. However, to our knowledge, there are no models of somitogenesis that explicitly consider the diffusion of protein and mRNA molecules within a cell. With a non-spatial modelling approach, it is impossible to answer simple but potentially important questions. For example, how fast do molecules move inside a cell? How does the shape of a cell or its nucleus, or the ratio of cell to nuclear size, influence the dynamics within a cell, or the signalling dynamics between cells? Hence, in this paper, we have derived a mathematical model of the zebrafish segmentation clock in which the diffusion of molecules is considered explicitly and in which the nucleus and cytoplasm are treated as separate compartments. Our model has focused on two adjacent cells in the presomitic mesoderm (PSM) which communicate by Notch signalling.

Our model, built on previous work by Lewis et al [1], [6], contains a negative feedback loop within each cell. We have found by simulation that this negative feedback loop can cause oscillatory dynamics to occur inside each cell. Moreover we have found that positive regulation of transcription due to Notch signalling can cause the oscillations in the two cells to synchronise. These results are consistent with the weight of experimental evidence that suggests the correct formation of somites is dependent on oscillatory gene expression in individual PSM cells with oscillations synchronised in neighbouring cells. An original feature of our work is that we have explicitly accounted for cell membrane interactions between Notch receptors and their DeltaC ligands, thereby also allowing us to model the release of Notch intracellular domain (NICD) into each cell. We achieved this by describing a binding rate function between the receptors and their ligands.

Our simulation results are robust to changes in the shape and size of the cells and their nuclei, a reassuring discovery given that biological pattern formation would be impossible without a certain robustness to cell shape and size. We have considered rounded, square, and hexagonal cell shapes, circular and elliptical nuclei, and different ratios of cell to nuclear size. Our explicitly spatial modelling approach allowed us to estimate the diffusion coefficients of the species in our model. We hope that our estimates will inspire others to measure these coefficients, thereby either confirming our estimates or providing new information by which we could improve our model. Certainly imaging technology has advanced in recent years, making it easier to measure diffusion rates.

We have found ranges of values for our model parameters such that oscillations synchronise in the two cells within a biologically reasonable timescale. These parameters include diffusion coefficients, degradation rates, translation rates, basal transcription rates, critical concentrations, the minimum distance of translation from the nucleus, and Hill coefficients. In particular, our model contains two Hill coefficients, one controlling the negative feedback role of Her1 and Her7 proteins, the other controlling the positive feedback of Notch signalling. The ranges of values for Hill coefficients that yield synchronised oscillations are narrow, indicating tight regulation of the feedback mechanisms. For the negative feedback Hill coefficient, synchronised oscillations are found only for the values 3 and 4, and for the positive feedback Hill coefficient, synchronised oscillations are found only for the value 1. In addition, we noticed during our simulation study that oscillations can synchronise in the two cells even when the diffusion coefficients in one cell are different to those in the other.

By plotting spatial profiles of the species in our model, we have gained insight into the distribution and interaction of these species. Animations provide further insight and are included as Supporting Information files (see Animation S2, S2, S3, and S4).

In order to reproduce experimental observations, models of intracellular oscillatory dynamics, including the model of Lewis upon which our work builds [1], [6], typically rely on a small number of time delays, representing various transport and interaction processes, that are incorporated into ordinary differential equations (ODEs) to create delay differential equations (DDEs). Our results show that using a model of diffusion using partial differential equations (PDEs) removes the need to rely on time delays in order to faithfully reproduce experimental observations. Moreover, models of PDEs enable us to account for the explicit movement of molecules or to study the spatial distribution of interacting species, neither of which is possible using systems of ODEs or DDEs, even when such systems model nuclear and cytoplasmic compartments separately [74], [75]. In addition, our PDE-based spatial model allowed us to control the distance from the nucleus at which proteins were synthesised in the cells; in essence, we were able to control the location of ribosomes.

Our spatial modelling approach can be applied to signalling pathways other than Notch-Delta. For example, we are currently applying it to the p53 pathway which regulates the cell cycle and is found to be de-regulated in 50% of human cancers [76]. An understanding of the spatial distribution of p53 within a cell is likely to be of value to clinicians seeking to treat cancer by targeted drug therapy.

Given that transcriptional control systems are subject to stochastic effects, we shall consider stochasticity in future work, for example by utilising the Gillespie algorithm [55]–[57]. In cells, molecules move not only by diffusion but can also be actively transported from a region of low concentration to a region of high concentration. In future research, we will incorporate active transport into our model. We also intend to further this work by examining a bigger range of cell geometries, for instance by looking at cell extensions, and by asking what happens when transcription occurs only at very specific locations (genes) in the nucleus, rather than at all points in the nucleus as assumed in our model.

There is evidence for oscillatory gene expression during somitogenesis in chicks and mice. Currently somitogenesis appears to be more clearly understood in zebrafish than in chicks or mice but in due course we envisage being able to write down mathematical models appropriate to these latter creatures. Our exploration in this paper has focused on the clock aspect of the “clock-and-wavefront” mechanism that has been used to describe somitogenesis. As future research, we may consider extending our model to contain a row of three or more cells, or a block of cells, through which there could diffuse a wavefront in the form of a morphogen gradient - for a given cell architecture, we could assume that a signalling molecule such as Wnt or Fibroblast Growth Factor could emanate from one end (the tail) of the architecture. Finally we may consider seeking analytical results for our model, exploiting symmetries to simplify this task.

## Methods

The simulations presented in this paper were produced using the COMSOL/FEMLAB package, which uses a finite element technique for solving partial differential equations. In all simulations, we used triangular basis elements, Lagrange quadratic basis functions, and a backward Euler time-stepping method of integration. Further details on COMSOL may be found online [77].

## Supporting Information

Text S1 This file includes the statement of the full system of equations for our core oscillator and Notch signalling models, details about their non-dimensionalisation, details about simulating these non-dimensionalised models, and calculations of parameter values in the dimensional models. In addition, there are figures demonstrating the robustness of our approach to changes in cell and nuclear shape.
(PDF)

Animation S1 Animation of *her1* mRNA concentration created by a numerical simulation of the non-dimensionalised core oscillator model, with parameters as in figure 3. Time and concentration are shown in non-dimensional units.
(MP4)

Animation S2 Animation of Her1 protein concentration created by a numerical simulation of the non-dimensionalised core oscillator model, with parameters as in figure 3. Time and concentration are shown in non-dimensional units.
(MP4)

Animation S3 Animation of *her1* mRNA concentration created by a numerical simulation of the non-dimensionalised Notch signalling model, with parameters as in the middle plot in figure 7. Time and concentration are shown in non-dimensional units.
(MP4)

Animation S4 Animation of Her1 protein concentration created by a numerical simulation of the non-dimensionalised Notch signalling model, with parameters as in the middle plot in figure 7. Time and concentration are shown in non-dimensional units.
(MP4)
