# Supplementary material for: A Spatio-Temporal Model of Notch Signalling in the Zebrafish Segmentation Clock: Conditions for Synchronised Oscillatory Dynamics
Source: PLoS One. 2011 Feb 28;6(2):e16980. doi: 10.1371/journal.pone.0016980 (PMC3046134; doi:10.1371/journal.pone.0016980)
Supplement: Text S1 — This file includes the statement of the full system of equations for our core oscillator and Notch signalling models, details about their non-dimensionalisation, details about simulating these non-dimensionalised models, and calculations of parameter values in the dimensional models. In addition, there are figures demonstrating the robustness of our approach to changes in cell and nuclear shape. (PDF) [file pone.0016980.s001.pdf]

Supporting Information file: Text S1

Alan J. Terry[†]*, Marc Sturrock[†], J. Kim Dale[‡], Miguel Maroto[‡], and Mark A. J. Chaplain[†]

[†]Division of Mathematics, University of Dundee, Dundee, United Kingdom

[‡]Division of Cell and Developmental Biology, University of Dundee, Dundee, United Kingdom

*Corresponding author
E-mail: aterry@maths.dundee.ac.uk

1 Introduction

This document contains data supplemental to the paper "A spatio-temporal model of Notch signalling in the zebrafish segmentation clock: conditions for synchronised oscillatory dynamics" by Alan J. Terry et al, which we henceforth refer to simply as "the paper".

2 The core oscillator

The core oscillator model is described in section 2.2 of the paper but not all of the equations are stated explicitly. We state the full set of equations in the current section.

The core oscillator model is a system of partial differential equations (PDEs) with two independent space variables, x and y, and the independent time variable t. It is defined on two regions, namely the nucleus and cytoplasm of a presomitic mesoderm (PSM) cell in a zebrafish.

To fully describe the model, we must define dependent variables:

- $[p_{\mathrm{her1}}(x, y, t)]$, $[p_{\mathrm{her7}}(x, y, t)]$, and $[p_{\mathrm{deltaC}}(x, y, t)]$ are, respectively, the concentrations at time t of the Her1, Her7, and DeltaC proteins at the point (x, y) in the cell
- $[m_{\mathrm{her1}}(x, y, t)]$, $[m_{\mathrm{her7}}(x, y, t)]$, and $[m_{\mathrm{deltaC}}(x, y, t)]$ are, respectively, the concentrations at time t of *her1* mRNA, *her7* mRNA, and *deltaC* mRNA at the point (x, y) in the cell.

For ease of notation, we omit unnecessary reference to the independent variables henceforth. Thus, for example, we will refer to $[p_{\mathrm{her1}}(x, y, t)]$ simply as $[p_{\mathrm{her1}}]$, and so on.

Define the following model parameters:

- D_K is the diffusion coefficient for the chemical species K; thus, for example, D_{pher1} is the diffusion coefficient for $[p_{\mathrm{her1}}]$, the Her1 protein
- a_{her1}, a_{her7}, and a_{deltaC} are, respectively, the translation rates per *her1*, *her7*, and *deltaC* mRNA molecule
- b_K is the degradation rate per molecule for the chemical species K; thus, for example, b_{pher1} is the degradation rate for $[p_{\mathrm{her1}}]$, the Her1 protein

- A is the minimum distance from the nucleus where translation can occur; then, if r is the distance of a point (x, y) in the cytoplasm from the nucleus, we define $\theta_r = 0$ when $r < A$ and $\theta_r = 1$ otherwise

- k_{her1}, k_{her7}, and k_{deltaC} are the basal transcription rates (or transcription rates in the absence of inhibition from the Her1 and Her7 proteins) of *her1*, *her7*, and *deltaC* mRNA, respectively

- $p_{0\mathrm{her}}$ is the concentration of the Her1 and Her7 proteins that reduces the transcription rates of the *her1*, *her7*, and *deltaC* mRNAs to half their basal values

- n is a Hill coefficient that determines the strength of the inhibitory action of Her1 and Her7.

We are now in a position to define the model. Reasoning as in section 2.2 of the paper, we suppose in the cytoplasm that

$$\frac{\partial [p_{\mathrm{her1}}]}{\partial t} = D_{\mathrm{pher1}} \nabla^2 [p_{\mathrm{her1}}] + \theta_r a_{\mathrm{her1}} [m_{\mathrm{her1}}] - b_{\mathrm{pher1}} [p_{\mathrm{her1}}] \quad (1)$$

$$\frac{\partial [p_{\mathrm{her7}}]}{\partial t} = D_{\mathrm{pher7}} \nabla^2 [p_{\mathrm{her7}}] + \theta_r a_{\mathrm{her7}} [m_{\mathrm{her7}}] - b_{\mathrm{pher7}} [p_{\mathrm{her7}}] \quad (2)$$

$$\frac{\partial [p_{\mathrm{deltaC}}]}{\partial t} = D_{\mathrm{pdeltaC}} \nabla^2 [p_{\mathrm{deltaC}}] + \theta_r a_{\mathrm{deltaC}} [m_{\mathrm{deltaC}}] - b_{\mathrm{pdeltaC}} [p_{\mathrm{deltaC}}] \quad (3)$$

$$\frac{\partial [m_{\mathrm{her1}}]}{\partial t} = D_{\mathrm{mher1}} \nabla^2 [m_{\mathrm{her1}}] - b_{\mathrm{mher1}} [m_{\mathrm{her1}}] \quad (4)$$

$$\frac{\partial [m_{\mathrm{her7}}]}{\partial t} = D_{\mathrm{mher7}} \nabla^2 [m_{\mathrm{her7}}] - b_{\mathrm{mher7}} [m_{\mathrm{her7}}] \quad (5)$$

$$\frac{\partial [m_{\mathrm{deltaC}}]}{\partial t} = D_{\mathrm{mdeltaC}} \nabla^2 [m_{\mathrm{deltaC}}] - b_{\mathrm{mdeltaC}} [m_{\mathrm{deltaC}}], \quad (6)$$

and in the nucleus we suppose that

$$\frac{\partial [p_{\mathrm{her1}}]}{\partial t} = D_{\mathrm{pher1}} \nabla^2 [p_{\mathrm{her1}}] - b_{\mathrm{pher1}} [p_{\mathrm{her1}}] \quad (7)$$

$$\frac{\partial [p_{\mathrm{her7}}]}{\partial t} = D_{\mathrm{pher7}} \nabla^2 [p_{\mathrm{her7}}] - b_{\mathrm{pher7}} [p_{\mathrm{her7}}] \quad (8)$$

$$\frac{\partial [m_{\mathrm{her1}}]}{\partial t} = D_{\mathrm{mher1}} \nabla^2 [m_{\mathrm{her1}}] + \frac{k_{\mathrm{her1}}}{1 + \left(\frac{[p_{\mathrm{her1}}]}{p_{0\mathrm{her}}}\right)^n \left(\frac{[p_{\mathrm{her7}}]}{p_{0\mathrm{her}}}\right)^n} - b_{\mathrm{mher1}} [m_{\mathrm{her1}}] \quad (9)$$

$$\frac{\partial [m_{\mathrm{her7}}]}{\partial t} = D_{\mathrm{mher7}} \nabla^2 [m_{\mathrm{her7}}] + \frac{k_{\mathrm{her7}}}{1 + \left(\frac{[p_{\mathrm{her1}}]}{p_{0\mathrm{her}}}\right)^n \left(\frac{[p_{\mathrm{her7}}]}{p_{0\mathrm{her}}}\right)^n} - b_{\mathrm{mher7}} [m_{\mathrm{her7}}] \quad (10)$$

$$\frac{\partial [m_{\mathrm{deltaC}}]}{\partial t} = D_{\mathrm{mdeltaC}} \nabla^2 [m_{\mathrm{deltaC}}] + \frac{k_{\mathrm{deltaC}}}{1 + \left(\frac{[p_{\mathrm{her1}}]}{p_{0\mathrm{her}}}\right)^n \left(\frac{[p_{\mathrm{her7}}]}{p_{0\mathrm{her}}}\right)^n} - b_{\mathrm{mdeltaC}} [m_{\mathrm{deltaC}}]. \quad (11)$$

In order to have a well-defined model, we require initial conditions and boundary conditions. These are stated in section 2.2 of the paper but we repeat them here for ease of reference. As in the DDE model of Lewis [1, 2], we choose zero initial conditions, that is, we choose all initial mRNA and protein concentrations in both the nucleus and cytoplasm to be zero. In terms of boundary conditions, we choose zero flux at the cell membrane, ensuring that no molecules are exported across it.

At the nuclear membrane we choose zero flux for the DeltaC protein to prevent it from entering the nucleus. For all other variables, we choose continuity of flux at the nuclear membrane, allowing import into and export out of the nucleus.

We can state our boundary conditions more explicitly. If we let m denote either m_{her1}, m_{her7}, or m_{deltaC}, and let the subscripts n and c denote nuclear and cytoplasmic concentrations respectively, then at the nuclear membrane

$$D_m \frac{\partial [m_n]}{\partial \mathbf{n}} = D_m \frac{\partial [m_c]}{\partial \mathbf{n}} \quad \text{and} \quad [m_n] = [m_c], \tag{12}$$

and at the cell membrane

$$\frac{\partial [m_c]}{\partial \mathbf{n}} = 0, \tag{13}$$

where $\mathbf{n}$ is a unit normal.

Similarly, if we let p denote either p_{her1} or p_{her7}, then at the nuclear membrane

$$D_p \frac{\partial [p_n]}{\partial \mathbf{n}} = D_p \frac{\partial [p_c]}{\partial \mathbf{n}} \quad \text{and} \quad [p_n] = [p_c], \tag{14}$$

and at the cell membrane

$$\frac{\partial [p_c]}{\partial \mathbf{n}} = 0. \tag{15}$$

Finally, if we let p denote p_{deltaC}, then at the nuclear and cell membranes

$$\frac{\partial [p_c]}{\partial \mathbf{n}} = 0. \tag{16}$$

2.1 Non-dimensionalisation

The model described in the previous section (the dimensional core oscillator model) shall be non-dimensionalised in this subsection. The virtue of non-dimensionalising has been described in section 2.2.1 of the paper. To non-dimensionalise our model, we first define reference values:

- reference concentrations: p_{ref} for proteins and m_{ref} for mRNAs
- reference time: τ (chosen to make the oscillatory period in the dimensional model equal the experimentally observed oscillatory period)
- reference length: L (chosen to make the cell in the dimensional model the same size as a zebrafish PSM cell).

Next we define re-scaled variables. In the following, re-scaled variables have tildes:

$$[p_{\text{her1}}] = [\tilde{p}_{\text{her1}}]\, p_{\text{ref}}, \quad [p_{\text{her7}}] = [\tilde{p}_{\text{her7}}]\, p_{\text{ref}}, \quad [p_{\text{deltaC}}] = [\tilde{p}_{\text{deltaC}}]\, p_{\text{ref}}, \tag{17}$$

$$[m_{\text{her1}}] = [\tilde{m}_{\text{her1}}]\, m_{\text{ref}}, \quad [m_{\text{her7}}] = [\tilde{m}_{\text{her7}}]\, m_{\text{ref}}, \quad [m_{\text{deltaC}}] = [\tilde{m}_{\text{deltaC}}]\, m_{\text{ref}}, \tag{18}$$

$$t = \tilde{t}\tau, \qquad x = \tilde{x}L, \qquad y = \tilde{y}L. \tag{19}$$

The parameters in the dimensional core oscillator model (section 2) are independent of the dependent and independent variables, except for A which represents a distance from the nucleus and therefore depends on the independent spatial variables, x and y. We define a re-scaled parameter A^* by requiring it to satisfy the following equation:

$$A = A^* L. \tag{20}$$

Using the re-scaling in (17) to (20) as well as the chain rule for differentation, we obtain from the dimensional model (equations (1) to (11)) a non-dimensionalised model. In this non-dimensionalised model, we find in the cytoplasm that

$$\frac{\partial\left[\tilde{p}_{\text{her1}}\right]}{\partial\tilde{t}} = D^*_{\text{pher1}}\nabla^2\left[\tilde{p}_{\text{her1}}\right] + \theta^*_r a^*_{\text{her1}}\left[\tilde{m}_{\text{her1}}\right] - b^*_{\text{pher1}}\left[\tilde{p}_{\text{her1}}\right] \tag{21}$$

$$\frac{\partial\left[\tilde{p}_{\text{her7}}\right]}{\partial\tilde{t}} = D^*_{\text{pher7}}\nabla^2\left[\tilde{p}_{\text{her7}}\right] + \theta^*_r a^*_{\text{her7}}\left[\tilde{m}_{\text{her7}}\right] - b^*_{\text{pher7}}\left[\tilde{p}_{\text{her7}}\right] \tag{22}$$

$$\frac{\partial\left[\tilde{p}_{\text{deltaC}}\right]}{\partial\tilde{t}} = D^*_{\text{pdeltaC}}\nabla^2\left[\tilde{p}_{\text{deltaC}}\right] + \theta^*_r a^*_{\text{deltaC}}\left[\tilde{m}_{\text{deltaC}}\right] - b^*_{\text{pdeltaC}}\left[\tilde{p}_{\text{deltaC}}\right] \tag{23}$$

$$\frac{\partial\left[\tilde{m}_{\text{her1}}\right]}{\partial\tilde{t}} = D^*_{\text{mher1}}\nabla^2\left[\tilde{m}_{\text{her1}}\right] - b^*_{\text{mher1}}\left[\tilde{m}_{\text{her1}}\right] \tag{24}$$

$$\frac{\partial\left[\tilde{m}_{\text{her7}}\right]}{\partial\tilde{t}} = D^*_{\text{mher7}}\nabla^2\left[\tilde{m}_{\text{her7}}\right] - b^*_{\text{mher7}}\left[\tilde{m}_{\text{her7}}\right] \tag{25}$$

$$\frac{\partial\left[\tilde{m}_{\text{deltaC}}\right]}{\partial\tilde{t}} = D^*_{\text{mdeltaC}}\nabla^2\left[\tilde{m}_{\text{deltaC}}\right] - b^*_{\text{mdeltaC}}\left[\tilde{m}_{\text{deltaC}}\right], \tag{26}$$

and in the nucleus we find that

$$\frac{\partial\left[\tilde{p}_{\text{her1}}\right]}{\partial\tilde{t}} = D^*_{\text{pher1}}\nabla^2\left[\tilde{p}_{\text{her1}}\right] - b^*_{\text{pher1}}\left[\tilde{p}_{\text{her1}}\right] \tag{27}$$

$$\frac{\partial\left[\tilde{p}_{\text{her7}}\right]}{\partial\tilde{t}} = D^*_{\text{pher7}}\nabla^2\left[\tilde{p}_{\text{her7}}\right] - b^*_{\text{pher7}}\left[\tilde{p}_{\text{her7}}\right] \tag{28}$$

$$\frac{\partial\left[\tilde{m}_{\text{her1}}\right]}{\partial\tilde{t}} = D^*_{\text{mher1}}\nabla^2\left[\tilde{m}_{\text{her1}}\right] + \frac{k^*_{\text{her1}}}{1+\left(\frac{\left[\tilde{p}_{\text{her1}}\right]}{p^*_{0\text{her}}}\right)^n\left(\frac{\left[\tilde{p}_{\text{her7}}\right]}{p^*_{0\text{her}}}\right)^n} - b^*_{\text{mher1}}\left[\tilde{m}_{\text{her1}}\right] \tag{29}$$

$$\frac{\partial\left[\tilde{m}_{\text{her7}}\right]}{\partial\tilde{t}} = D^*_{\text{mher7}}\nabla^2\left[\tilde{m}_{\text{her7}}\right] + \frac{k^*_{\text{her7}}}{1+\left(\frac{\left[\tilde{p}_{\text{her1}}\right]}{p^*_{0\text{her}}}\right)^n\left(\frac{\left[\tilde{p}_{\text{her7}}\right]}{p^*_{0\text{her}}}\right)^n} - b^*_{\text{mher7}}\left[\tilde{m}_{\text{her7}}\right] \tag{30}$$

$$\frac{\partial\left[\tilde{m}_{\text{deltaC}}\right]}{\partial\tilde{t}} = D^*_{\text{mdeltaC}}\nabla^2\left[\tilde{m}_{\text{deltaC}}\right] + \frac{k^*_{\text{deltaC}}}{1+\left(\frac{\left[\tilde{p}_{\text{her1}}\right]}{p^*_{0\text{her}}}\right)^n\left(\frac{\left[\tilde{p}_{\text{her7}}\right]}{p^*_{0\text{her}}}\right)^n} - b^*_{\text{mdeltaC}}\left[\tilde{m}_{\text{deltaC}}\right], \tag{31}$$

where

$$D^*_{\text{pher1}} = \frac{D_{\text{pher1}}\tau}{L^2}, \quad D^*_{\text{pher7}} = \frac{D_{\text{pher7}}\tau}{L^2}, \quad D^*_{\text{pdeltaC}} = \frac{D_{\text{pdeltaC}}\tau}{L^2}, \tag{32}$$

$$D^*_{\text{mher1}} = \frac{D_{\text{mher1}}\tau}{L^2}, \quad D^*_{\text{mher7}} = \frac{D_{\text{mher7}}\tau}{L^2}, \quad D^*_{\text{mdeltaC}} = \frac{D_{\text{mdeltaC}}\tau}{L^2}, \tag{33}$$

$$a^*_{\text{her1}} = \frac{a_{\text{her1}}m_{\text{ref}}\tau}{p_{\text{ref}}}, \quad a^*_{\text{her7}} = \frac{a_{\text{her7}}m_{\text{ref}}\tau}{p_{\text{ref}}}, \quad a^*_{\text{deltaC}} = \frac{a_{\text{deltaC}}m_{\text{ref}}\tau}{p_{\text{ref}}}, \tag{34}$$

$$b^*_{\text{pher1}} = b_{\text{pher1}}\tau, \quad b^*_{\text{pher7}} = b_{\text{pher7}}\tau, \quad b^*_{\text{pdeltaC}} = b_{\text{pdeltaC}}\tau, \tag{35}$$

$$b^*_{\text{mher1}} = b_{\text{mher1}}\tau, \quad b^*_{\text{mher7}} = b_{\text{mher7}}\tau, \quad b^*_{\text{mdeltaC}} = b_{\text{mdeltaC}}\tau, \tag{36}$$

$$k^*_{\text{her1}} = \frac{k_{\text{her1}}\tau}{m_{\text{ref}}}, \quad k^*_{\text{her7}} = \frac{k_{\text{her7}}\tau}{m_{\text{ref}}}, \quad k^*_{\text{deltaC}} = \frac{k_{\text{deltaC}}\tau}{m_{\text{ref}}}, \tag{37}$$

$$p^*_{0\text{her}} = \frac{p_{0\text{her}}}{p_{\text{ref}}}, \tag{38}$$

and where $\theta_r^* = 0$ if $r < A^*$ and $\theta_r^* = 1$ otherwise, where distance is measured in the transformed variables, $\tilde{x}$ and $\tilde{y}$.

The initial conditions and boundary conditions are unchanged by non-dimensionalising: we retain zero initial conditions, zero flux boundary conditions at the cell membrane, zero flux for $\tilde{p}_{\text{deltaC}}$ at the nuclear membrane, and continuity of flux for the other species at the nuclear membrane. Hence, the conditions stated in equations (12) to (16) still hold, except that D_m, m_n, m_c, D_p, p_n, and p_c are replaced in these equations by D_m^*, $\tilde{m}_n$, $\tilde{m}_c$, D_p^*, $\tilde{p}_n$, and $\tilde{p}_c$ respectively.

2.2 Simulations

To carry out simulations in the non-dimensionalised core oscillator model (equations (21) to (31)), we must choose a geometry on which the model equations hold as well as values for the model parameters. Our aim is to make choices that yield oscillatory dynamics. Fortunately there is no need for us to simply guess a geometry or parameter values; we can allow our choices to be influenced by a recent study by Sturrock et al [3] of the Hes1 protein, which is known to play a role in somitogenesis in mice [4]. Sturrock et al consider a PDE model similar to our core oscillator model; for a non-dimensionalised version of their model, they find a geometry and parameter values that generate sustained oscillations in Hes1 concentration. By choosing a geometry with similar size and identical or similar parameter values in our non-dimensionalised core oscillator model, we have obtained oscillatory dynamics.

The geometry on which we define our non-dimensionalised core oscillator model is:

- a circle, radius 0.4, centre $(0, 0)$, which represents the nucleus
- a hexagon, with corners (in clockwise order) at $(-1, -0.6)$, $(-1, 0.6)$, $(0, 1.2)$, $(1, 0.6)$, $(1, -0.6)$, and $(0, -1.2)$, which (minus the circle defined in the previous bullet point) represents the cytoplasm.

We depict this geometry in figure 1. We chose a hexagonal cell shape because zebrafish PSM cells are often this shape, though they can also be approximately rectangular or rounded [5].

In order to discuss parameter values that yield oscillatory dynamics, it is useful to make some simplifying assumptions. Specifically, assume that all diffusion coefficients are equal, all degradation rates are equal, all translation rates are equal, and all basal transcription rates are equal:

$$D^*_{\text{pher1}} = D^*_{\text{pher7}} = D^*_{\text{pdeltaC}} = D^*_{\text{mher1}} = D^*_{\text{mher7}} = D^*_{\text{mdeltaC}} = D^*, \qquad (39)$$

$$b^*_{\text{pher1}} = b^*_{\text{pher7}} = b^*_{\text{pdeltaC}} = b^*_{\text{mher1}} = b^*_{\text{mher7}} = b^*_{\text{mdeltaC}} = b^*, \qquad (40)$$

$$a^*_{\text{her1}} = a^*_{\text{her7}} = a^*_{\text{deltaC}} = a^*, \qquad k^*_{\text{her1}} = k^*_{\text{her7}} = k^*_{\text{deltaC}} = k^*, \qquad (41)$$

where D^*, b^*, a^*, and k^* are positive constants. Given that the different mRNA and protein species in the core oscillator all seem to be controlled by the same negative feedback loop, it is not necessarily inappropriate to suggest, for example, that they have the same diffusion coefficient or the same degradation rate. Indeed, Lewis simulated his DDE model by assuming, for example, that all degradation rates were identical in [1] or almost identical in work with Ozbudak in [2].

We find oscillatory dynamics (with period 193 non-dimensional time units, simulating up to $\tilde{t} = 6000$) for the following parameter choices:

$$D^* = 0.00075, \; b^* = 0.03, \; a^* = 1, \; k^* = 1, \; p^*_{0\text{her}} = 1, \; A^* = 0.1, \; n = 3. \qquad (42)$$

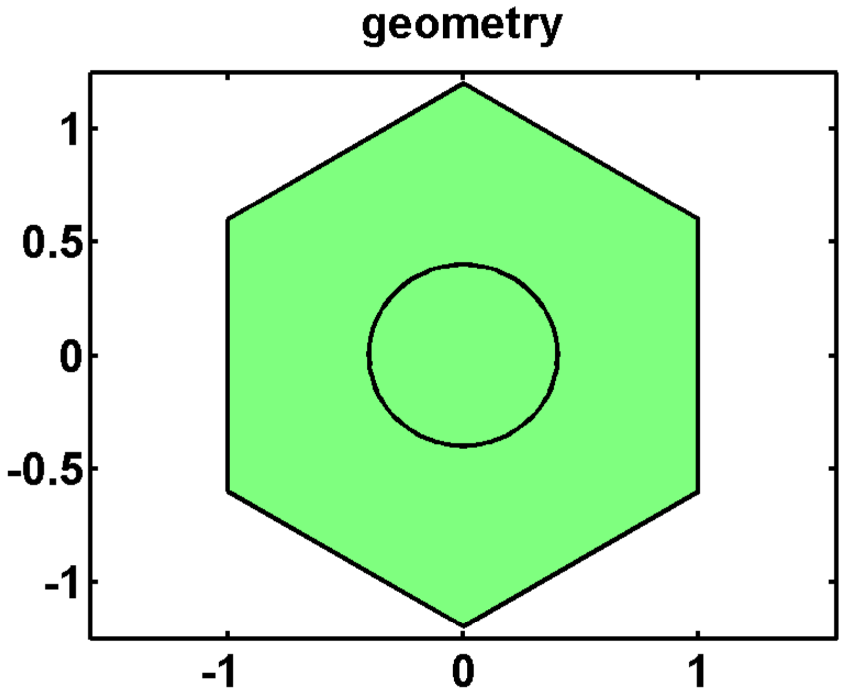

Figure 1: The non-dimensionalised core oscillator model (equations (21) to (31)) holds on the green geometry shown here. The geometry is a hexagonal cell with two compartments, the nucleus (the area within the circle) and the cytoplasm.

Figures 3, 4, and 5 in the paper are simulations of the non-dimensionalised core oscillator model (with time rescaled to fit the experimentally observed oscillatory period of 30 minutes [1]) where the geometry is defined as in figure 1 and the parameter values are chosen as in (39) to (42). We have found that oscillations are robust to changes in cell geometry (see figures 2 and 3).

The Hill coefficient in our dimensionalised model is the same as in our non-dimensionalised model. Although Lewis uses a Hill coefficient of $n = 1$ in his DDE model [1], we were unable to find sustained oscillatory dynamics in our non-dimensionalised model for $n = 1$. We have found sustained oscillatory dynamics for $n = 2$ (for example, with the parameter choices $D^* = 0.0005$, $b^* = 0.025$, $a^* = 2$, $k^* = 1$, $p^*_{0\text{her}} = 1$, $A^* = 0.1$) but oscillations appear to be less robust to changes in the parameters for $n = 2$ than for $n = 3$ (results not shown). For simplicity we have investigated only integer Hill coefficients; discussion of the meaning and use of non-integer Hill coefficients may be found in [4, 6].

We can calculate parameter values that generate sustained oscillations in the dimensional model (equations (1) to (11)). To do this, we first estimate the reference time τ and reference length L. As noted in section 2.2.1 of the paper, the reference time τ is chosen to make the oscillatory period in the dimensional model equal the experimentally observed oscillatory period. Now we have said that the oscillatory period in our simulations is 193 non-dimensional time units. But by (19), we have $t = \tilde{t}\tau$, so 1 non-dimensional time unit is τ dimensional time units. Hence the oscillatory period in the dimensional model is 193τ dimensional time units. We set the oscillatory period in the dimensional model equal to the experimentally observed oscillatory period, which is 30 minutes or 1800 seconds [1], to obtain $193\tau = 1800\text{s}$, so that:

$$\tau = 9.33\text{s}. \tag{43}$$

As noted in section 2.2.1 of the paper, the reference length L is chosen to make the cell in the dimensional model the same size as a zebrafish PSM cell. The width of our non-dimensionalised cell is 2 non-dimensionalised spatial units (figure 1) or $2L$ dimensional spatial units (using (19)). Also, the diameter of a zebrafish PSM cell varies from around $10\mu\text{m}$ to $15\mu\text{m}$ [5], so we set $2L = 12\mu\text{m}$, which yields:

$$L = 6\mu\text{m}. \tag{44}$$

Given this value of L, we find that the nuclear diameter in our cell in dimensional spatial units is $0.8 \times 6\mu\text{m} = 4.8\mu\text{m}$. Such a value is very similar to the $5\mu\text{m}$ nuclear diameter for a zebrafish PSM cell mentioned in [1, 7]. We can trivially see that oscillations are robust to changes in the size of the cell if we simply change the size of the reference length L.

Using (20), (42), and (44), we find:

$$A = 0.6\mu\text{m}. \tag{45}$$

Using (32), (33), (39), (42), (43), and (44), we find:

$$\begin{aligned} &D_{\text{pher1}} = D_{\text{pher7}} = D_{\text{pdeltaC}} = D_{\text{mher1}} = D_{\text{mher7}} = D_{\text{mdeltaC}} \\ &= 0.0029\mu\text{m}^2\text{s}^{-1} = 2.9 \times 10^{-11}\text{cm}^2\text{s}^{-1}. \end{aligned} \tag{46}$$

Using (35), (36), (40), (42), and (43), we find:

$$b_{\text{pher1}} = b_{\text{pher7}} = b_{\text{pdeltaC}} = b_{\text{mher1}} = b_{\text{mher7}} = b_{\text{mdeltaC}} = 0.0032\text{s}^{-1}. \tag{47}$$

Our degradation rates estimate of 0.0032s^{-1} is pleasingly consistent with the estimate Lewis has made for the degradation rates, namely 0.23min^{-1} or 0.0038s^{-1} [1].

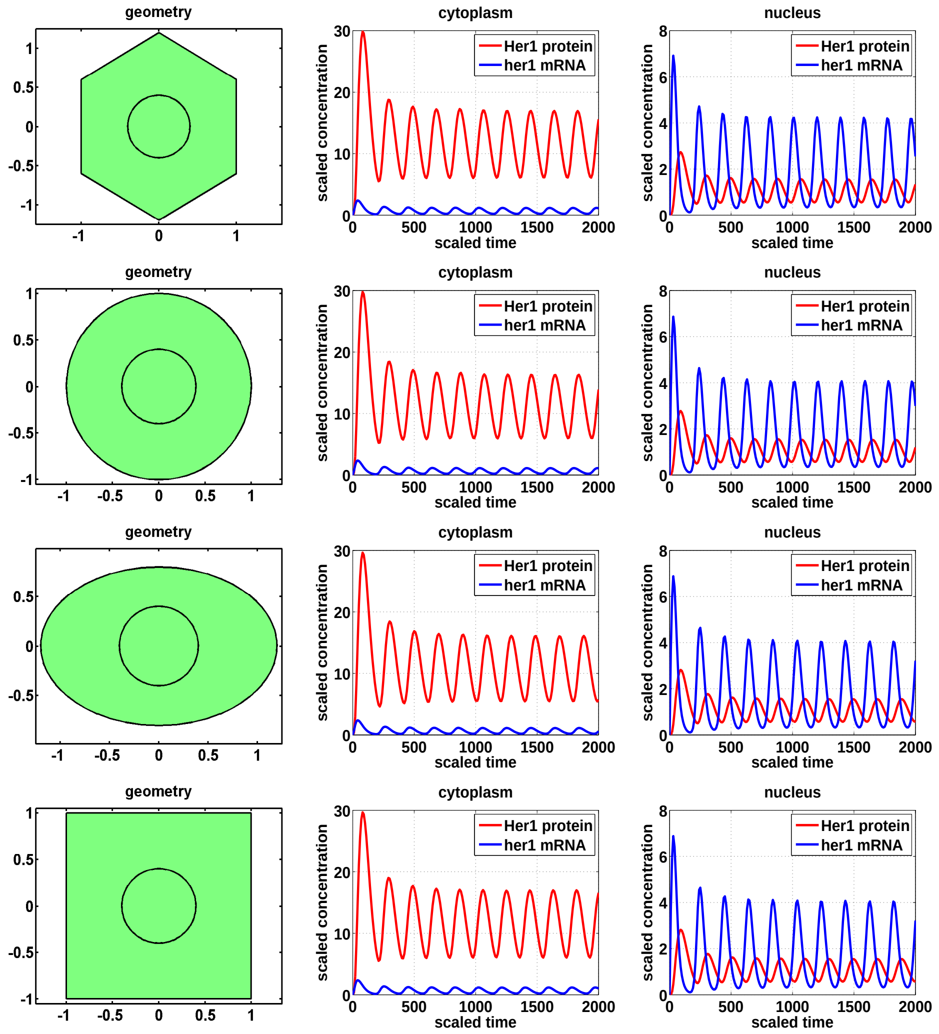

Figure 2: Simulations of the non-dimensionalised core oscillator model (equations (21) to (31)) with parameters determined by (39) to (42). Each row shows the geometry on which a simulation is performed, with the corresponding numerical results to the right of each geometry. The nucleus is the same in every geometry; here we vary only the shape of the cell. Oscillatory dynamics are clearly robust to this variation. Concentrations and times are shown in non-dimensional units.

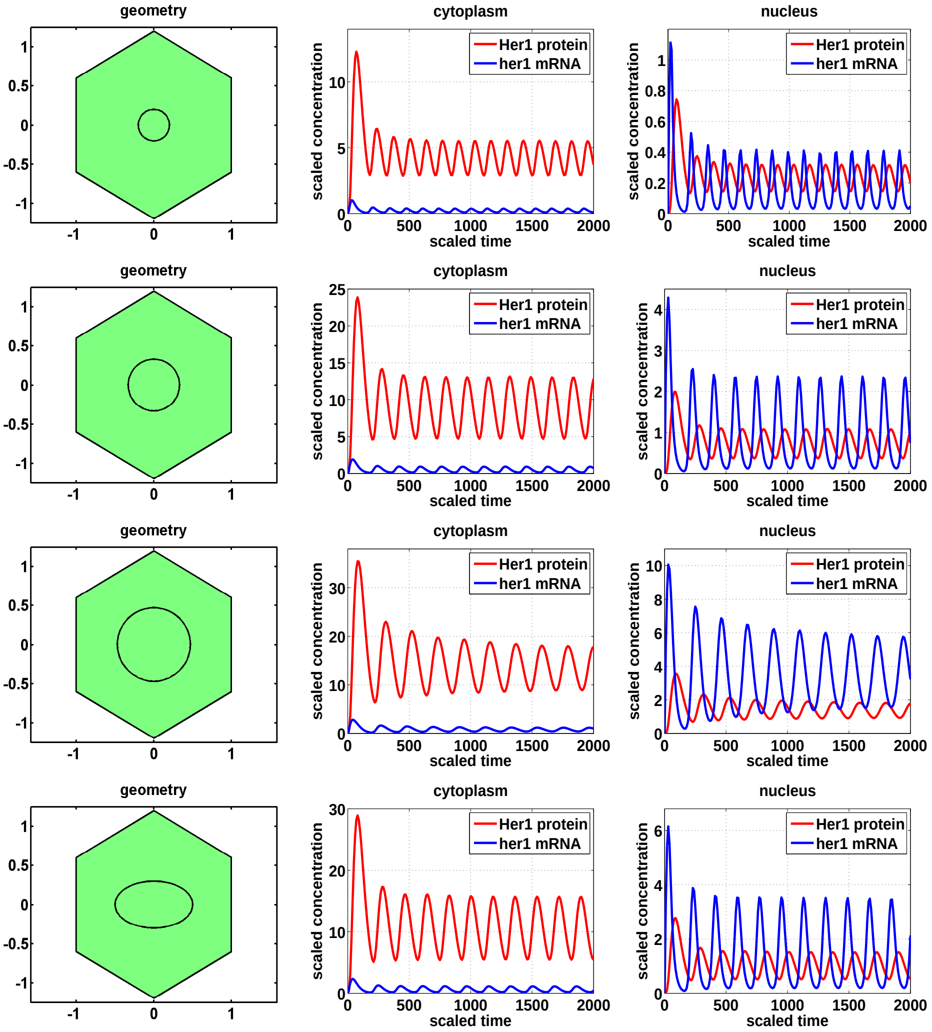

Figure 3: Simulations of the non-dimensionalised core oscillator model (equations (21) to (31)) with parameters determined by (39) to (42). Each row shows the geometry on which a simulation is performed, with the corresponding numerical results to the right of each geometry. The cell shape is the same in every geometry; here we vary only the size and shape of the nucleus. Oscillatory dynamics are robust to this variation, although the amplitude dies down somewhat when the nucleus is large (third row). Concentrations and times are shown in non-dimensional units.

The remaining dimensional parameters have values dependent on the reference concentrations, p_{ref} and m_{ref} (first mentioned in subsection 2.1). In fact, by (34), (41), and (42), we see that:

$$a_{\mathrm{her1}} = a_{\mathrm{her7}} = a_{\mathrm{deltaC}} = \frac{p_{\mathrm{ref}}}{9.33 m_{\mathrm{ref}}} \mathrm{s}^{-1}. \tag{48}$$

Also, by (37), (41), and (42), we see that:

$$k_{\mathrm{her1}} = k_{\mathrm{her7}} = k_{\mathrm{deltaC}} = \frac{m_{\mathrm{ref}}}{9.33} \mathrm{s}^{-1}. \tag{49}$$

Finally, by (38) and (42), we have:

$$p_{0\mathrm{her}} = p_{\mathrm{ref}}. \tag{50}$$

It is not obvious how to estimate the reference concentrations and fortunately we do not have to. Lewis has previously estimated the critical concentration $p_{0\mathrm{her}}$ to be 10^{-9}M within a 5μm diameter nucleus [1]. Our dimensional cell has a nuclear diameter of approximately 5μm, so we shall write:

$$p_{0\mathrm{her}} = 10^{-9}\mathrm{M}. \tag{51}$$

By (50) and (51), we clearly have:

$$p_{\mathrm{ref}} = 10^{-9}\mathrm{M}. \tag{52}$$

The translation rates (a_{her1}, a_{her7}, a_{deltaC}) have been estimated by Lewis at $4.5\mathrm{min}^{-1} = 0.075\mathrm{s}^{-1}$ [1]. Hence we assume that:

$$a_{\mathrm{her1}} = a_{\mathrm{her7}} = a_{\mathrm{deltaC}} = 0.075\mathrm{s}^{-1}. \tag{53}$$

By (48), (52), and (53), we have:

$$m_{\mathrm{ref}} = 1.43 \times 10^{-9}\mathrm{M}. \tag{54}$$

Then, by (49) and (54), we find:

$$k_{\mathrm{her1}} = k_{\mathrm{her7}} = k_{\mathrm{deltaC}} = 1.53 \times 10^{-10}\mathrm{Ms}^{-1}. \tag{55}$$

We have now calculated values for all dimensional parameters such that the dimensional model will yield oscillatory dynamics. However, we have found that sustained oscillations occur for ranges of parameter values. We describe our method for finding such ranges in the next subsection.

2.3 Parameter ranges yielding sustained oscillations

Consider the non-dimensionalised core oscillator model (equations (21) to (31)). If we make the simplifying assumptions in (39) to (41), then we reduce the number of model parameters, being left with the following: D^*, b^*, a^*, k^*, A^*, $p^*_{0\mathrm{her}}$, n. If we fix all but one of these parameters as in (42), then we can vary the parameter that has not been fixed to find a range of its values such that the model exhibits sustained oscillations (simulating on the geometry in figure 1 up to $\tilde{t} = 6000$, initial and boundary conditions as in the final paragraph of subsection 2.1). Performing a simulation study of this kind for each of the parameters allowed us to construct table 1. We note, for example, that for values of D^* closer to the extremes of the range stated in table 1, the amplitude of the oscillations becomes smaller and slightly erratic. For values of b^* towards the bottom of the range stated in table 1

Non-dimensional parameter	Range of values giving sustained oscillations
Diffusion coefficients D^* (all species)	0.0005 to 0.0025
Degradation rates b^* (all species)	0.0062 to 0.036
Translation rates a^* (of Her1, Her7, DeltaC proteins)	≥ 0.33
Basal transcription rates k^* (of *her1*, *her7*, *deltaC* mRNAs)	≥ 0.45
Minimal distance A^* of translation from nucleus	0 to 0.23
Critical concentration $p^*_{0\text{her}}$ of Her1 and Her7 proteins	0.0006 to 3.35
Hill coefficient n	≥ 3

Table 1: Ranges of parameter values in the non-dimensionalised core oscillator model (equations (21) to (31)) that yield sustained oscillatory dynamics (simulating on the geometry in figure 1 up to $\tilde{t} = 6000$ non-dimensional time units). To find each range, we first made the simplifying assumptions on the parameters in (39) to (41). Then we held all but one of the parameters fixed as in (42) and varied the remaining parameter to find its range of values yielding sustained oscillations.

there are very small amplitudes in the oscillations, whilst for values towards the top of this range the amplitudes are slightly erratic. A more detailed parameter sensitivity analysis for the models presented in this work will be considered as future work; our purpose in this subsection is to demonstrate how to find parameter ranges yielding oscillations.

We can convert the ranges for the non-dimensional parameters in table 1 into ranges for dimensional parameters. Assume that the reference time τ, reference length L, and reference concentrations, p_{ref} and m_{ref}, satisfy (43), (44), (52), and (54) respectively. Then, using equations (20), (32) to (38), and assumptions (39) to (41), we readily obtain by trivial calculations the dimensional parameter ranges in table 1 in the paper.

3 Notch signalling model

The Notch signalling model is described in section 2.3 of the paper but not all of the equations are not stated explicitly. We state the equations in the current section.

The Notch signalling model is a system of PDEs with two independent space variables, x and y, and the independent time variable t. It is defined on two cells, cells 1 and 2, which represent neighbouring zebrafish PSM cells. There are five regions on which our model is defined, namely the nucleus and cytoplasm of cell 1, the nucleus and cytoplasm of cell 2, and the region where the cell membranes of the two cells touch, which we shall refer to as the membrane subdomain.

To describe the model, we must first define dependent variables. For $i = 1, 2$, then, we suppose:

- $[p_{\text{her1}}(x, y, t)]_i$, $[p_{\text{her7}}(x, y, t)]_i$, and $[p_{\text{deltaC}}(x, y, t)]_i$ are, respectively, the concentrations at time t of the Her1, Her7, and DeltaC proteins at the point (x, y) in cell i

- $[m_{\text{her1}}(x,y,t)]_i$, $[m_{\text{her7}}(x,y,t)]_i$, and $[m_{\text{deltaC}}(x,y,t)]_i$ are, respectively, the concentrations at time t of *her1* mRNA, *her7* mRNA, and *deltaC* mRNA at the point (x,y) in cell i
- $[m_{\text{notch}}(x,y,t)]_i$ and $[p_{\text{notch}}(x,y,t)]_i$ are, respectively, the concentrations at time t of *notch* mRNA and Notch protein (receptors) at the point (x,y) in cell i
- $[\text{NICD}(x,y,t)]_i$ is the concentration at time t of Notch intracellular domain (NICD) at the point (x,y) in cell i.

For ease of notation, we henceforth omit unnecessary reference to the independent variables. Thus, for example, we will refer to $[p_{\text{her1}}(x,y,t)]_i$ simply as $[p_{\text{her1}}]_i$, and so on.

Define model parameters as in the core oscillator model (section 2). Assume that the model parameters in cell 1 are the same as those in cell 2. Hence, for example, D_{pher1} is the diffusion coefficient for p_{her1}, the Her1 protein, in both cells 1 and 2. In addition, define parameters as follows:

- D_{mnotch}, D_{pnotch}, and D_{NICD} are, respectively, the diffusion coefficients for *notch* mRNA, Notch protein, and NICD
- b_{mnotch}, b_{pnotch}, and b_{NICD} are, respectively, the degradation rates per *notch* mRNA, Notch protein, and NICD molecule
- a_{notch} is the translation rate per *notch* mRNA molecule
- k_{notch} is the transcription rate of *notch* mRNA
- M_0 is a critical concentration of Notch above which there is sufficient Notch for signalling in cell i to be mostly dependent on DeltaC from cell j $(1 \leq i \neq j \leq 2)$
- p_{0d} is a critical concentration of DeltaC such that NICD release in cell i is higher for DeltaC from cell j above p_{0d} in the membrane subdomain $(1 \leq i \neq j \leq 2)$
- β is half the rate at which $[\text{NICD}]_i$ is released in the membrane subdomain if $[p_{\text{deltaC}}]_j = p_{0d}$ and $[p_{\text{notch}}]_i = M_0$ in the membrane subdomain $(1 \leq i \neq j \leq 2)$
- m is a positive constant that plays a role akin to a Hill coefficient for transcriptional upregulation by NICD
- N_0 is a critical concentration of NICD above which its upregulatory impact on transcription is stronger.

We can now write down the PDEs that hold in the membrane subdomain:

$$\frac{\partial [p_{\text{deltaC}}]_i}{\partial t} = D_{\text{pdeltaC}} \nabla^2 [p_{\text{deltaC}}]_i - \frac{\beta \left(\frac{[p_{\text{deltaC}}]_i}{p_{0d}}\right)\left(\frac{[p_{\text{notch}}]_j}{M_0}\right)}{1+\left(\frac{[p_{\text{notch}}]_j}{M_0}\right)} - b_{\text{pdeltaC}} [p_{\text{deltaC}}]_i \tag{56}$$

$$\frac{\partial [p_{\text{notch}}]_i}{\partial t} = D_{\text{pnotch}} \nabla^2 [p_{\text{notch}}]_i - \frac{\beta \left(\frac{[p_{\text{deltaC}}]_j}{p_{0d}}\right)\left(\frac{[p_{\text{notch}}]_i}{M_0}\right)}{1+\left(\frac{[p_{\text{notch}}]_i}{M_0}\right)} - b_{\text{pnotch}} [p_{\text{notch}}]_i \tag{57}$$

$$\frac{\partial [\text{NICD}]_i}{\partial t} = D_{\text{NICD}} \nabla^2 [\text{NICD}]_i + \frac{\beta \left(\frac{[p_{\text{deltaC}}]_j}{p_{0d}}\right)\left(\frac{[p_{\text{notch}}]_i}{M_0}\right)}{1+\left(\frac{[p_{\text{notch}}]_i}{M_0}\right)} - b_{\text{NICD}} [\text{NICD}]_i \,, \tag{58}$$

where $1 \leq i \neq j \leq 2$.

For $i = 1, 2$, the PDEs in the cytoplasm for cell i are as follows:

$$\frac{\partial [p_{\text{notch}}]_i}{\partial t} = D_{\text{pnotch}} \nabla^2 [p_{\text{notch}}]_i + \theta_r a_{\text{notch}} [m_{\text{notch}}]_i - b_{\text{pnotch}} [p_{\text{notch}}]_i \quad (59)$$

$$\frac{\partial [m_{\text{notch}}]_i}{\partial t} = D_{\text{mnotch}} \nabla^2 [m_{\text{notch}}]_i - b_{\text{mnotch}} [m_{\text{notch}}]_i \quad (60)$$

$$\frac{\partial [\text{NICD}]_i}{\partial t} = D_{\text{NICD}} \nabla^2 [\text{NICD}]_i - b_{\text{NICD}} [\text{NICD}]_i \quad (61)$$

$$\frac{\partial [p_{\text{her1}}]_i}{\partial t} = D_{\text{pher1}} \nabla^2 [p_{\text{her1}}]_i + \theta_r a_{\text{her1}} [m_{\text{her1}}]_i - b_{\text{pher1}} [p_{\text{her1}}]_i \quad (62)$$

$$\frac{\partial [p_{\text{her7}}]_i}{\partial t} = D_{\text{pher7}} \nabla^2 [p_{\text{her7}}]_i + \theta_r a_{\text{her7}} [m_{\text{her7}}]_i - b_{\text{pher7}} [p_{\text{her7}}]_i \quad (63)$$

$$\frac{\partial [p_{\text{deltaC}}]_i}{\partial t} = D_{\text{pdeltaC}} \nabla^2 [p_{\text{deltaC}}]_i + \theta_r a_{\text{deltaC}} [m_{\text{deltaC}}]_i - b_{\text{pdeltaC}} [p_{\text{deltaC}}]_i \quad (64)$$

$$\frac{\partial [m_{\text{her1}}]_i}{\partial t} = D_{\text{mher1}} \nabla^2 [m_{\text{her1}}]_i - b_{\text{mher1}} [m_{\text{her1}}]_i \quad (65)$$

$$\frac{\partial [m_{\text{her7}}]_i}{\partial t} = D_{\text{mher7}} \nabla^2 [m_{\text{her7}}]_i - b_{\text{mher7}} [m_{\text{her7}}]_i \quad (66)$$

$$\frac{\partial [m_{\text{deltaC}}]_i}{\partial t} = D_{\text{mdeltaC}} \nabla^2 [m_{\text{deltaC}}]_i - b_{\text{mdeltaC}} [m_{\text{deltaC}}]_i \,. \quad (67)$$

The PDEs that hold in the nucleus of cell i ($i = 1, 2$) are as follows:

$$\frac{\partial [m_{\text{notch}}]_i}{\partial t} = D_{\text{mnotch}} \nabla^2 [m_{\text{notch}}]_i + k_{\text{notch}} - b_{\text{mnotch}} [m_{\text{notch}}]_i \quad (68)$$

$$\frac{\partial [\text{NICD}]_i}{\partial t} = D_{\text{NICD}} \nabla^2 [\text{NICD}]_i - b_{\text{NICD}} [\text{NICD}]_i \quad (69)$$

$$\frac{\partial [p_{\text{her1}}]_i}{\partial t} = D_{\text{pher1}} \nabla^2 [p_{\text{her1}}]_i - b_{\text{pher1}} [p_{\text{her1}}]_i \quad (70)$$

$$\frac{\partial [p_{\text{her7}}]_i}{\partial t} = D_{\text{pher7}} \nabla^2 [p_{\text{her7}}]_i - b_{\text{pher7}} [p_{\text{her7}}]_i \quad (71)$$

$$\frac{\partial [m_{\text{her1}}]_i}{\partial t} = D_{\text{mher1}} \nabla^2 [m_{\text{her1}}]_i + \frac{k_{\text{her1}} \left(1 + \left(\frac{[\text{NICD}]_i}{N_0}\right)^m\right)}{1 + \left(\frac{[\text{NICD}]_i}{N_0}\right)^m + \left(\frac{[p_{\text{her1}}]_i}{p_{0\text{her}}}\right)^n \left(\frac{[p_{\text{her7}}]_i}{p_{0\text{her}}}\right)^n} - b_{\text{mher1}} [m_{\text{her1}}]_i \quad (72)$$

$$\frac{\partial [m_{\text{her7}}]_i}{\partial t} = D_{\text{mher7}} \nabla^2 [m_{\text{her7}}]_i + \frac{k_{\text{her7}} \left(1 + \left(\frac{[\text{NICD}]_i}{N_0}\right)^m\right)}{1 + \left(\frac{[\text{NICD}]_i}{N_0}\right)^m + \left(\frac{[p_{\text{her1}}]_i}{p_{0\text{her}}}\right)^n \left(\frac{[p_{\text{her7}}]_i}{p_{0\text{her}}}\right)^n} - b_{\text{mher7}} [m_{\text{her7}}]_i \quad (73)$$

$$\frac{\partial [m_{\text{deltaC}}]_i}{\partial t} = D_{\text{mdeltaC}} \nabla^2 [m_{\text{deltaC}}]_i + \frac{k_{\text{deltaC}}}{1 + \left(\frac{[p_{\text{her1}}]_i}{p_{0\text{her}}}\right)^n \left(\frac{[p_{\text{her7}}]_i}{p_{0\text{her}}}\right)^n} - b_{\text{mdeltaC}} [m_{\text{deltaC}}]_i \,. \quad (74)$$

For a well-defined model, we require initial conditions and boundary conditions. As in the DDE model of Lewis [1, 2], we choose zero initial conditions, that is, we choose all initial mRNA and protein concentrations to be zero. In terms of boundary conditions, we choose continuity of flux at the nuclear membrane in cell i ($i = 1, 2$) for all species defined in cell i, except for $[p_{\text{deltaC}}]_i$ and $[p_{\text{notch}}]_i$ for which we choose zero flux. These nuclear membrane boundary conditions allow import

and export of mRNA and protein across the nuclear membrane, except for $[p_{\text{deltaC}}]_i$ and $[p_{\text{notch}}]_i$ which are prevented from entering the nucleus.

At the border between cell i and the membrane subdomain, we choose zero flux for all species except for the variables $[p_{\text{deltaC}}]_i$, $[p_{\text{notch}}]_i$, and $[\text{NICD}]_i$ for which we choose continuity of flux. At all other cell membrane or membrane subdomain boundaries we choose zero flux for all variables. These boundary conditions for the cell membranes and the membrane subdomain ensure that no molecules except those involved in Notch signalling are exported across a cell membrane. Our choices for the boundary conditions are consistent with the modelling assumptions described in section 2.3 of the paper.

It may facilitate understanding to write out our boundary conditions more explicitly. If we let K denote any species (mRNA or protein) in cell i except DeltaC protein or Notch, and let the subscripts n and c denote nuclear and cytoplasmic concentrations respectively, then at the nuclear membrane,

$$D_K \frac{\partial [K_n]_i}{\partial \mathbf{n}} = D_K \frac{\partial [K_c]_i}{\partial \mathbf{n}} \quad \text{and} \quad [K_n]_i = [K_c]_i, \tag{75}$$

where $\mathbf{n}$ is a unit normal.

If we let K denote either DeltaC protein, Notch, or NICD in cell i, and let the subscripts c and m denote cytoplasmic and membrane subdomain concentrations respectively, then at the cell membrane between cell i and the membrane subdomain,

$$D_K \frac{\partial [K_c]_i}{\partial \mathbf{n}} = D_K \frac{\partial [K_m]_i}{\partial \mathbf{n}} \quad \text{and} \quad [K_c]_i = [K_m]_i. \tag{76}$$

If we let K denote any species (mRNA or protein) in cell i, then at all boundaries where the conditions described by (75) and (76) do not hold and where K may actually come into contact with the boundary,

$$\frac{\partial [K]_i}{\partial \mathbf{n}} = 0. \tag{77}$$

3.1 Non-dimensionalisation

To non-dimensionalise the Notch signalling model (equations (56) to (74)), we first define reference values:

- reference concentrations: p_{ref} for the Her1, Her7, and DeltaC proteins, m_{ref} for *her1*, *her7*, and *deltaC* mRNAs, $\text{pnotch}_{\text{ref}}$ for Notch, $\text{mnotch}_{\text{ref}}$ for notch mRNA, and NICD_{ref} for NICD
- reference time: τ (chosen to make the oscillatory period in the dimensional model equal the experimentally observed oscillatory period)
- reference length: L (chosen to make the cells in the dimensional model the same size as zebrafish PSM cells).

Next we introduce re-scaled variables. In the following, for $i = 1, 2$, re-scaled variables have tildes:

$$[m_{\text{notch}}]_i = [\tilde{m}_{\text{notch}}]_i \, \text{mnotch}_{\text{ref}}, \qquad [p_{\text{notch}}]_i = [\tilde{p}_{\text{notch}}]_i \, \text{pnotch}_{\text{ref}}, \tag{78}$$

$$[\text{NICD}]_i = \left[\widetilde{\text{NICD}}\right]_i \text{NICD}_{\text{ref}}, \tag{79}$$

$$[p_{\text{her1}}]_i = [\tilde{p}_{\text{her1}}]_i \, p_{\text{ref}}, \qquad [p_{\text{her7}}]_i = [\tilde{p}_{\text{her7}}]_i \, p_{\text{ref}}, \qquad [p_{\text{deltaC}}]_i = [\tilde{p}_{\text{deltaC}}]_i \, p_{\text{ref}}, \tag{80}$$

$$[m_{\text{her1}}]_i = [\tilde{m}_{\text{her1}}]_i \, m_{\text{ref}}, \quad [m_{\text{her7}}]_i = [\tilde{m}_{\text{her7}}]_i \, m_{\text{ref}}, \quad [m_{\text{deltaC}}]_i = [\tilde{m}_{\text{deltaC}}]_i \, m_{\text{ref}}, \tag{81}$$

$$t = \tilde{t}\tau, \qquad x = \tilde{x}L, \qquad y = \tilde{y}L. \tag{82}$$

The parameters in the dimensional Notch signalling model (section 3) are independent of the dependent and independent variables, except for A which represents a distance from the nucleus and therefore depends on the independent spatial variables, x and y. We define a re-scaled parameter A^* by requiring it to satisfy the following equation:

$$A = A^* L. \tag{83}$$

Using the re-scaling in (78) to (83) as well as the chain rule for differentation, we obtain from the dimensional model (equations (56) to (74)) a non-dimensionalised model. In this non-dimensionalised model, we obtain, for $1 \leq i \neq j \leq 2$, in the membrane subdomain,

$$\frac{\partial \left[\tilde{p}_{\text{deltaC}}\right]_i}{\partial \tilde{t}} = D^*_{\text{pdeltaC}} \nabla^2 \left[\tilde{p}_{\text{deltaC}}\right]_i - \frac{\beta_1^* \left(\frac{[\tilde{p}_{\text{deltaC}}]_i}{p^*_{0d}}\right)\left(\frac{[\tilde{p}_{\text{notch}}]_j}{M_0^*}\right)}{1 + \left(\frac{[\tilde{p}_{\text{notch}}]_j}{M_0^*}\right)} - b^*_{\text{pdeltaC}} \left[\tilde{p}_{\text{deltaC}}\right]_i \tag{84}$$

$$\frac{\partial \left[\tilde{p}_{\text{notch}}\right]_i}{\partial \tilde{t}} = D^*_{\text{pnotch}} \nabla^2 \left[\tilde{p}_{\text{notch}}\right]_i - \frac{\beta_2^* \left(\frac{[\tilde{p}_{\text{deltaC}}]_j}{p^*_{0d}}\right)\left(\frac{[\tilde{p}_{\text{notch}}]_i}{M_0^*}\right)}{1 + \left(\frac{[\tilde{p}_{\text{notch}}]_i}{M_0^*}\right)} - b^*_{\text{pnotch}} \left[\tilde{p}_{\text{notch}}\right]_i \tag{85}$$

$$\frac{\partial \left[\tilde{\text{NICD}}\right]_i}{\partial \tilde{t}} = D^*_{\text{NICD}} \nabla^2 \left[\tilde{\text{NICD}}\right]_i + \frac{\beta_3^* \left(\frac{[\tilde{p}_{\text{deltaC}}]_j}{p^*_{0d}}\right)\left(\frac{[\tilde{p}_{\text{notch}}]_i}{M_0^*}\right)}{1 + \left(\frac{[\tilde{p}_{\text{notch}}]_i}{M_0^*}\right)} - b^*_{\text{NICD}} \left[\tilde{\text{NICD}}\right]_i, \tag{86}$$

and we obtain, in the cytoplasm,

$$\frac{\partial \left[\tilde{p}_{\text{notch}}\right]_i}{\partial t} = D^*_{\text{pnotch}} \nabla^2 \left[\tilde{p}_{\text{notch}}\right]_i + \theta_r^* a^*_{\text{notch}} \left[\tilde{m}_{\text{notch}}\right]_i - b^*_{\text{pnotch}} \left[\tilde{p}_{\text{notch}}\right]_i \tag{87}$$

$$\frac{\partial \left[\tilde{m}_{\text{notch}}\right]_i}{\partial t} = D^*_{\text{mnotch}} \nabla^2 \left[\tilde{m}_{\text{notch}}\right]_i - b^*_{\text{mnotch}} \left[\tilde{m}_{\text{notch}}\right]_i \tag{88}$$

$$\frac{\partial \left[\tilde{\text{NICD}}\right]_i}{\partial \tilde{t}} = D^*_{\text{NICD}} \nabla^2 \left[\tilde{\text{NICD}}\right]_i - b^*_{\text{NICD}} \left[\tilde{\text{NICD}}\right]_i \tag{89}$$

$$\frac{\partial \left[\tilde{p}_{\text{her1}}\right]_i}{\partial \tilde{t}} = D^*_{\text{pher1}} \nabla^2 \left[\tilde{p}_{\text{her1}}\right]_i + \theta_r^* a^*_{\text{her1}} \left[\tilde{m}_{\text{her1}}\right]_i - b^*_{\text{pher1}} \left[\tilde{p}_{\text{her1}}\right]_i \tag{90}$$

$$\frac{\partial \left[\tilde{p}_{\text{her7}}\right]_i}{\partial \tilde{t}} = D^*_{\text{pher7}} \nabla^2 \left[\tilde{p}_{\text{her7}}\right]_i + \theta_r^* a^*_{\text{her7}} \left[\tilde{m}_{\text{her7}}\right]_i - b^*_{\text{pher7}} \left[\tilde{p}_{\text{her7}}\right]_i \tag{91}$$

$$\frac{\partial \left[\tilde{p}_{\text{deltaC}}\right]_i}{\partial \tilde{t}} = D^*_{\text{pdeltaC}} \nabla^2 \left[\tilde{p}_{\text{deltaC}}\right]_i + \theta_r^* a^*_{\text{deltaC}} \left[\tilde{m}_{\text{deltaC}}\right]_i - b^*_{\text{pdeltaC}} \left[\tilde{p}_{\text{deltaC}}\right]_i \tag{92}$$

$$\frac{\partial \left[\tilde{m}_{\text{her1}}\right]_i}{\partial \tilde{t}} = D^*_{\text{mher1}} \nabla^2 \left[\tilde{m}_{\text{her1}}\right]_i - b^*_{\text{mher1}} \left[\tilde{m}_{\text{her1}}\right]_i \tag{93}$$

$$\frac{\partial \left[\tilde{m}_{\text{her7}}\right]_i}{\partial \tilde{t}} = D^*_{\text{mher7}} \nabla^2 \left[\tilde{m}_{\text{her7}}\right]_i - b^*_{\text{mher1}} \left[\tilde{m}_{\text{her7}}\right]_i \tag{94}$$

$$\frac{\partial \left[\tilde{m}_{\text{deltaC}}\right]_i}{\partial \tilde{t}} = D^*_{\text{mdeltaC}} \nabla^2 \left[\tilde{m}_{\text{deltaC}}\right]_i - b^*_{\text{mdeltaC}} \left[\tilde{m}_{\text{deltaC}}\right]_i, \tag{95}$$

and finally we obtain, in the nucleus,

$$\frac{\partial\left[\tilde{m}_{\text{notch}}\right]_i}{\partial t} = D^*_{\text{mnotch}}\nabla^2\left[\tilde{m}_{\text{notch}}\right]_i + k^*_{\text{notch}} - b^*_{\text{mnotch}}\left[\tilde{m}_{\text{notch}}\right]_i \tag{96}$$

$$\frac{\partial\left[\widetilde{\text{NICD}}\right]_i}{\partial\tilde{t}} = D^*_{\text{NICD}}\nabla^2\left[\widetilde{\text{NICD}}\right]_i - b^*_{\text{NICD}}\left[\widetilde{\text{NICD}}\right]_i \tag{97}$$

$$\frac{\partial\left[\tilde{p}_{\text{her1}}\right]_i}{\partial\tilde{t}} = D^*_{\text{pher1}}\nabla^2\left[\tilde{p}_{\text{her1}}\right]_i - b^*_{\text{pher1}}\left[\tilde{p}_{\text{her1}}\right]_i \tag{98}$$

$$\frac{\partial\left[\tilde{p}_{\text{her7}}\right]_i}{\partial\tilde{t}} = D^*_{\text{pher7}}\nabla^2\left[\tilde{p}_{\text{her7}}\right]_i - b^*_{\text{pher7}}\left[\tilde{p}_{\text{her7}}\right]_i \tag{99}$$

$$\frac{\partial\left[\tilde{m}_{\text{her1}}\right]_i}{\partial\tilde{t}} = D^*_{\text{mher1}}\nabla^2\left[\tilde{m}_{\text{her1}}\right]_i + \frac{k^*_{\text{her1}}\left(1+\left(\frac{\left[\widetilde{\text{NICD}}\right]_i}{N_0^*}\right)^m\right)}{1+\left(\frac{\left[\widetilde{\text{NICD}}\right]_i}{N_0^*}\right)^m+\left(\frac{\left[\tilde{p}_{\text{her1}}\right]_i}{p^*_{0\text{her}}}\right)^n\left(\frac{\left[\tilde{p}_{\text{her7}}\right]_i}{p^*_{0\text{her}}}\right)^n} - b^*_{\text{mher1}}\left[\tilde{m}_{\text{her1}}\right] \tag{100}$$

$$\frac{\partial\left[\tilde{m}_{\text{her7}}\right]_i}{\partial\tilde{t}} = D^*_{\text{mher7}}\nabla^2\left[\tilde{m}_{\text{her7}}\right]_i + \frac{k^*_{\text{her7}}\left(1+\left(\frac{\left[\widetilde{\text{NICD}}\right]_i}{N_0^*}\right)^m\right)}{1+\left(\frac{\left[\widetilde{\text{NICD}}\right]_i}{N_0^*}\right)^m+\left(\frac{\left[\tilde{p}_{\text{her1}}\right]_i}{p^*_{0\text{her}}}\right)^n\left(\frac{\left[\tilde{p}_{\text{her7}}\right]_i}{p^*_{0\text{her}}}\right)^n} - b^*_{\text{mher7}}\left[\tilde{m}_{\text{her7}}\right]_i \tag{101}$$

$$\frac{\partial\left[\tilde{m}_{\text{deltaC}}\right]_i}{\partial\tilde{t}} = D^*_{\text{mdeltaC}}\nabla^2\left[\tilde{m}_{\text{deltaC}}\right]_i + \frac{k^*_{\text{deltaC}}}{1+\left(\frac{\left[\tilde{p}_{\text{her1}}\right]_i}{p^*_{0\text{her}}}\right)^n\left(\frac{\left[\tilde{p}_{\text{her7}}\right]_i}{p^*_{0\text{her}}}\right)^n} - b^*_{\text{mdeltaC}}\left[\tilde{m}_{\text{deltaC}}\right]_i, \tag{102}$$

where equations (32) to (38) hold, where

$$D^*_{\text{pnotch}} = \frac{D_{\text{pnotch}}\tau}{L^2}, \qquad D^*_{\text{mnotch}} = \frac{D_{\text{mnotch}}\tau}{L^2}, \qquad D^*_{\text{NICD}} = \frac{D_{\text{NICD}}\tau}{L^2}, \tag{103}$$

$$b^*_{\text{pnotch}} = b_{\text{pnotch}}\tau, \qquad b^*_{\text{mnotch}} = b_{\text{mnotch}}\tau, \qquad b^*_{\text{NICD}} = b_{\text{NICD}}\tau, \tag{104}$$

$$a^*_{\text{notch}} = \frac{a_{\text{notch}}\text{mnotch}_{\text{ref}}\tau}{\text{pnotch}_{\text{ref}}}, \qquad k^*_{\text{notch}} = \frac{k_{\text{notch}}\tau}{\text{mnotch}_{\text{ref}}}, \tag{105}$$

$$\beta^*_1 = \frac{\beta\tau}{\text{p}_{\text{ref}}}, \qquad \beta^*_2 = \frac{\beta\tau}{\text{pnotch}_{\text{ref}}}, \qquad \beta^*_3 = \frac{\beta\tau}{\text{NICD}_{\text{ref}}}, \tag{106}$$

$$p^*_{0d} = \frac{p_{0d}}{\text{p}_{\text{ref}}}, \qquad N^*_0 = \frac{N_0}{\text{NICD}_{\text{ref}}}, \qquad M^*_0 = \frac{M_0}{\text{pnotch}_{\text{ref}}}, \tag{107}$$

and where $\theta^*_r = 0$ if $r < A^*$ and $\theta^*_r = 1$ otherwise, where distance is measured in the transformed variables, $\tilde{x}$ and $\tilde{y}$.

Our assumptions for the initial and boundary conditions for the Notch signalling model are unchanged by non-dimensionalising. Hence, in particular, the conditions stated in equations (75) to (77) still hold where the species represented in these conditions are non-dimensionalised concentrations and the diffusion coefficients are also non-dimensionalised.

3.2 Simulations

To carry out simulations in the non-dimensionalised Notch signalling model (equations (84) to (102)), we must choose a geometry on which the model equations hold as well as values for the model parameters. Our aim is to make choices that yield oscillatory dynamics in each cell which synchronise due to the Notch signalling.

We have already defined a geometry and obtained oscillatory dynamics in our non-dimensionalised core oscillator model (subsection 2.2). The Notch signalling model is simply an extension of the core oscillator model, with the core oscillator mechanism holding in a slighly modified form in the two cells. It is therefore natural to use the same scale for the geometry and the same model parameters for each cell in the Notch signalling model that we used in the single cell in the core oscillator model. Hence the geometry on which we define our non-dimensionalised Notch signalling model is:

- a circle, radius 0.4, centre $(0, 0)$, which represents the nucleus in cell 1
- a hexagon, with corners (in clockwise order) at $(-1, -0.6)$, $(-1, 0.6)$, $(0, 1.2)$, $(1, 0.6)$, $(1, -0.6)$, and $(0, -1.2)$, which (minus the circle defined in the previous bullet point) represents the cytoplasm in cell 1
- a thin rectangle, with corners $(1, -0.6)$, $(1, 0.6)$, $(1.02, 0.6)$, and $(1.02, -0.6)$, which represents the touching cell membranes and which we call the membrane subdomain
- a circle, radius 0.4, centre $(2.02, 0)$, which represents the nucleus in cell 2
- a hexagon, with corners (in clockwise order) at $(1.02, -0.6)$, $(1.02, 0.6)$, $(2.02, 1.2)$, $(3.02, 0.6)$, $(3.02, -0.6)$, and $(2.02, -1.2)$, which (minus the circle defined in the previous bullet point) represents the cytoplasm in cell 2.

We depict this geometry in figure 4.

In choosing parameters with which to simulate the non-dimensionalised core oscillator model, we made several simplifying assumptions (equations (39) to (41)). We make these same assumptions in simulating our non-dimensionalised Notch signalling model, as well as the following assumptions:

$$D^*_{\text{mnotch}} = D^*_{\text{pnotch}} = D^*_{\text{NICD}} = D^*, \tag{108}$$

$$b^*_{\text{mnotch}} = b^*_{\text{pnotch}} = b^*_{\text{NICD}} = b^*, \tag{109}$$

$$a^*_{\text{notch}} = a^*, \tag{110}$$

where D^*, b^*, and a^* are the positive constants used in equations (39) to (41).

We found oscillatory dynamics in the non-dimensionalised core oscillator model for the parameter choices listed in (42). We make these same parameter choices in simulating the non-dimensionalised Notch signalling model. Then we find that Notch signalling synchronises the oscillations in the two cells when we additionally make the following parameter choices:

$$\beta^*_1 = \beta^*_2 = \beta^*_3 = m = k^*_{\text{notch}} = p^*_{0d} = 1, \qquad M^*_0 = 0.001, \qquad N^*_0 = 0.0001. \tag{111}$$

Using these parameter values and the geometry defined above, we created figure 7 (middle plot), figure 8, figure 9, figure 10 (middle plot), figure 11, and figure 12 in the paper (with time rescaled in these figures to fit the experimentally observed oscillatory period). In these figures we forced the two cells to begin completely out of synchrony by allowing transcription to occur from time $\tilde{t} = 0$ in cell 1 but preventing transcription from occurring in cell 2 until $\tilde{t} = 96.5$ ($t = 15$ minutes in dimensional time units) when half the oscillatory period for the core oscillator had passed. We controlled the start time of transcription in cell 2 using a Heaviside function (an inbuilt feature of COMSOL). We controlled the time that signalling began ($\tilde{t} = 965$ or $t = 150$ minutes) by using a Heaviside function to keep at zero the levels of NICD for both cells in the membrane subdomain before this time.

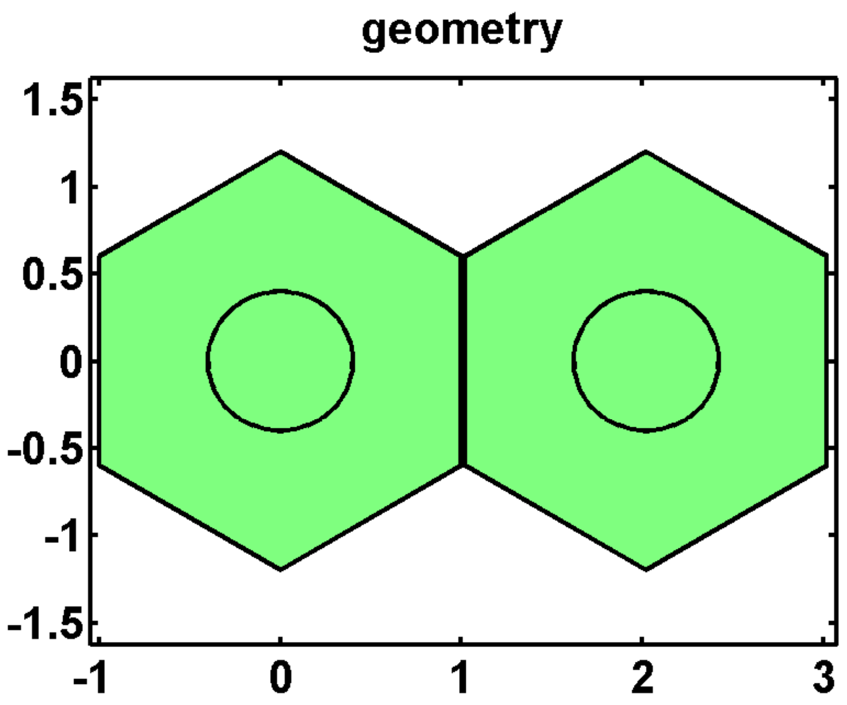

Figure 4: The geometry (shown in green) on which the non-dimensionalised Notch signalling model holds (equations (84) to (102)). There are five regions shown here. Cell 1, on the left, consists of a nucleus and cytoplasm. Cell 2, on the right, also consists of a nucleus and cytoplasm. In addition, there is a thin strip between the two cells, representing touching cell membranes and which we call the membrane subdomain. The membrane subdomain is sufficiently thin that it is not easy to discern in this figure.

Figure 7 (top) in the paper was created in the same way as figure 7 (middle) except that no signalling was allowed for the entire duration of the simulation. Oscillations in the neighbouring cells begin out of synchrony and, since the two cells are identical and there is no signalling, remain perfectly out of synchrony for the whole simulation. Figure 7 (bottom) in the paper was created in the same way as figure 7 (middle), except that we set $N_0^* = 0.00001$, causing the Notch signalling to have a bigger upregulatory impact on transcription (recall the definition of N_0 in section 3 and recall that N_0^* is proportional to N_0 by (107)).

For those parameter choices used to create figure 7 (middle) in the paper (the choices in (42) and (111)), we have found that the oscillatory period of the core oscillator in each cell is not significantly changed by synchronisation. We have also found that synchronisation will still occur when minor changes are made to the geometry (figures 5 to 7), and we have found that synchronisation can occur when the two cells have different parameter values which would cause them to have different oscillatory periods in the absence of signalling (figures 8 and 9).

In subsection 2.2 we calculated parameter values that yield oscillatory dynamics in the dimensional core oscillator model. Using the same approach, we can calculate parameter values that synchronise oscillations in the dimensional Notch signalling model (equations (56) to (74)). Let us do this where the non-dimensional parameter choices are as in (42) and (111). The Hill coefficients n and m in the dimensionalised Notch signalling model are the same as in the non-dimensionalised Notch signalling model (so $n = 3$ and $m = 1$). Our choice of $m = 1$ is consistent with the DDE model of Lewis, updated by Ozbudak and Lewis [2].

Let us estimate the reference time τ and reference length L. Since the oscillatory period is not significantly changed by synchronisation, and since our scale for the geometry of the non-dimensionalised Notch signalling model is the same (per cell) as in the non-dimensionalised core oscillator model, we can use the same reference time and reference length that we used in calculating parameter values for the dimensional core oscillator model, specifically the time and length stated in equations (43) and (44) respectively. (We can clearly see that synchronised oscillations are robust to changes in the sizes of the cells if we simply change the size of the reference length). Reasoning as in subsection 2.2 and also using (103), (104), (108), and (109), it follows trivially that

$$A = 0.6\mu\text{m}, \tag{112}$$

$$\begin{aligned} &D_{\text{pher1}} = D_{\text{pher7}} = D_{\text{pdeltaC}} = D_{\text{mher1}} = D_{\text{mher7}} = D_{\text{mdeltaC}} = D_{\text{pnotch}} \\ &= D_{\text{mnotch}} = D_{\text{NICD}} = 0.0029\mu\text{m}^2\text{s}^{-1} = 2.9 \times 10^{-11}\text{cm}^2\text{s}^{-1}, \end{aligned} \tag{113}$$

$$\begin{aligned} &b_{\text{pher1}} = b_{\text{pher7}} = b_{\text{pdeltaC}} = b_{\text{mher1}} = b_{\text{mher7}} = b_{\text{mdeltaC}} = b_{\text{pnotch}} \\ &= b_{\text{mnotch}} = b_{\text{NICD}} = 0.0032\text{s}^{-1}. \end{aligned} \tag{114}$$

We suppose that the critical concentration $p_{0\text{her}}$ satisfies (51), which again leads to p_{ref} satisfying (52). For ease of reference, we write out our values for $p_{0\text{her}}$ and p_{ref} again here:

$$p_{0\text{her}} = 10^{-9}\text{M}, \tag{115}$$

$$p_{\text{ref}} = 10^{-9}\text{M}. \tag{116}$$

By (107), (111), and (116), we have:

$$p_{0d} = p_{0d}^* p_{\text{ref}} = 10^{-9}\text{M}. \tag{117}$$

We assume the translation rates (a_{her1}, a_{her7}, a_{deltaC}) satisfy (53), which we repeat here:

$$a_{\text{her1}} = a_{\text{her7}} = a_{\text{deltaC}} = 0.075\text{s}^{-1}. \tag{118}$$

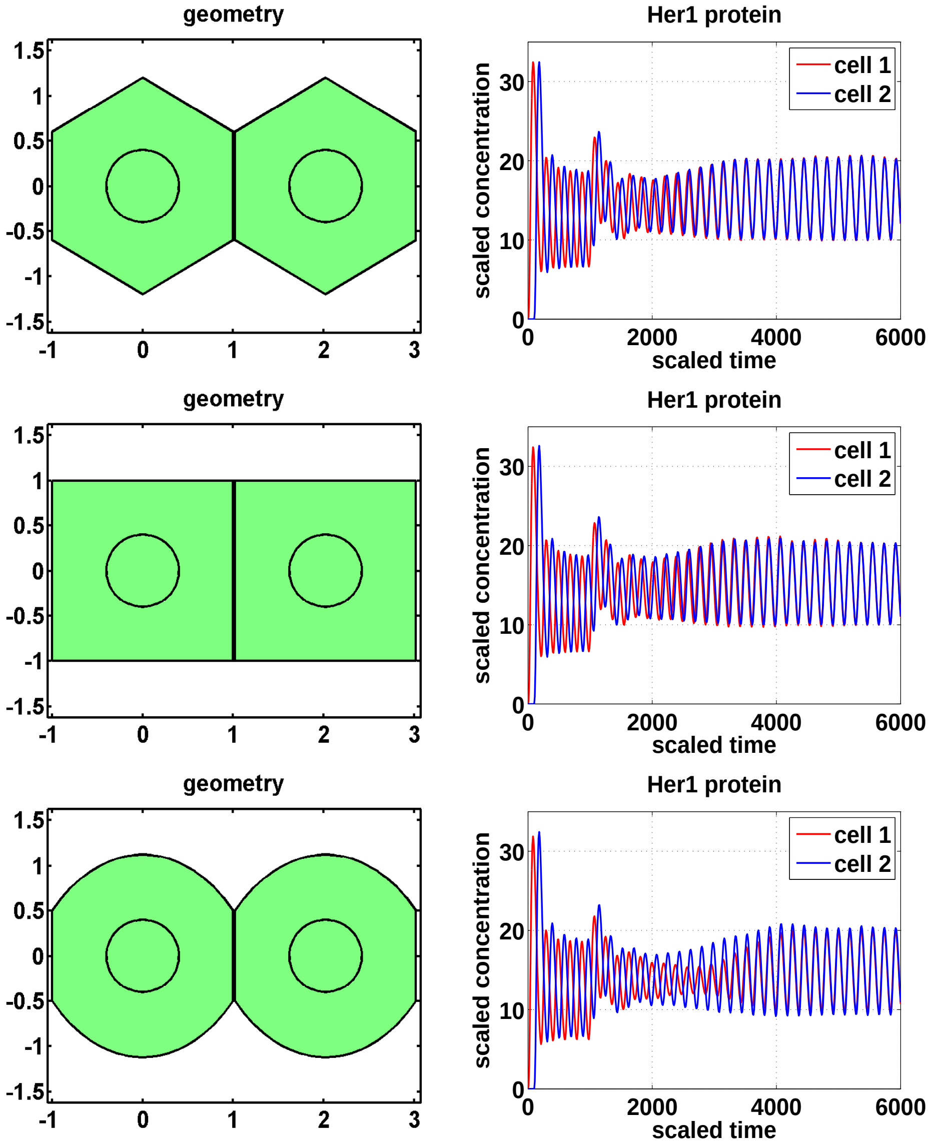

Figure 5: Simulations of the non-dimensionalised Notch signalling model (equations (84) to (102)) with parameters determined by (39) to (42) and by (108) to (111). Each row shows the geometry on which a simulation is performed, with the corresponding numerical results to the right of each geometry. The nuclei are the same in every geometry; here we vary only the shape of the cells. Transcription begins at time $\tilde{t} = 0$ in cell 1 and at time $\tilde{t} = 96.5$ non-dimensional units ($t = 15$ minutes in dimensional time units) in cell 2. Signalling begins at 965 non-dimensional time units (150 minutes); synchronisation is clearly robust to the geometrical variation presented here. Concentrations and times are shown in non-dimensional units.

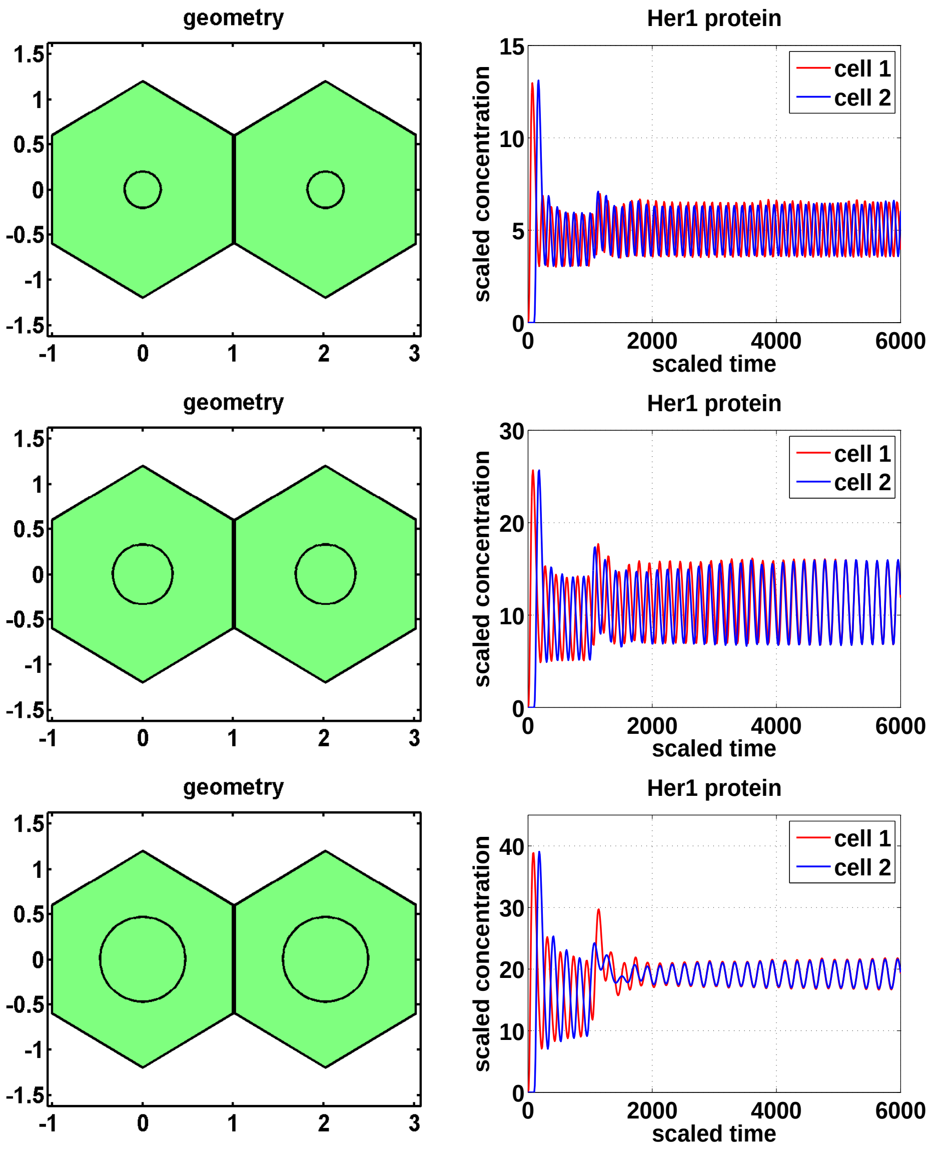

Figure 6: Simulations created in the same way as those in figure 5 except that here we vary the size of the nuclei and keep the cell shapes the same in every geometry. Both oscillatory dynamics and synchronisation are sensitive to this geometrical variation. For a nuclear radius of 0.32 or less, synchrony does not occur by time $\tilde{t} = 6000$. This situation is represented in the first row of plots here where the nuclear radius is 0.2. For a nuclear radius from 0.33 to 0.47, synchrony does occur. This is represented in the second row (nuclear radius 0.33) and third row (nuclear radius 0.47). For a nuclear radius of 0.48 or above, synchrony occurs but oscillations die out (result not shown). Concentrations and times are shown in non-dimensional units.

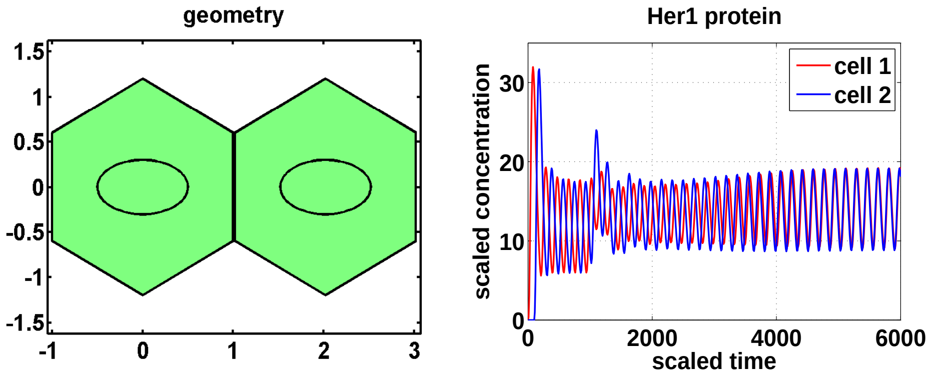

Figure 7: Plots created in the same way as those in the first row of figure 5 except that here we change the shape of the nuclei to ellipses (the nuclei have width 1.0 non-dimensional spatial units and height 0.6 non-dimensional spatial units). Synchronisation is robust to this change. Concentrations and times are shown in non-dimensional units.

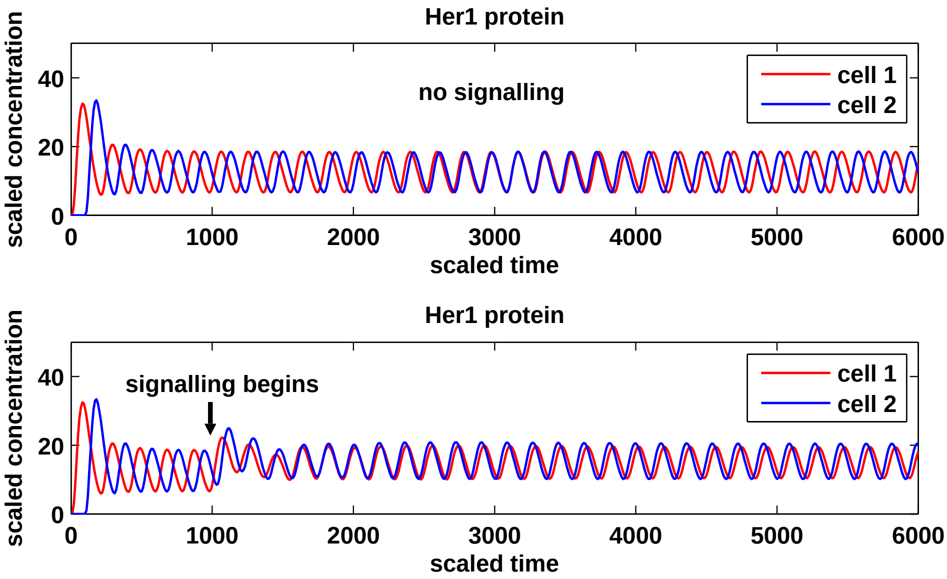

Figure 8: Simulations of the non-dimensionalised Notch signalling model (equations (84) to (102)) with parameters determined in cell 1 by (39) to (42) and by (108) to (111). The parameters in cell 2 are determined in the same way except that the diffusion coefficients are all increased by 10% relative to cell 1; thus, the non-dimensional diffusion rates are 0.00075 in cell 1 and 0.000825 in cell 2. In both plots, transcription begins at time $\tilde{t} = 0$ in cell 1 and at time $\tilde{t} = 96.5$ non-dimensional units ($t = 15$ minutes in dimensional time units) in cell 2, and the geometry is as shown in figure 4. In the top plot, there is no signalling; the cells drift in and out of synchrony. In the bottom plot, signalling begins at 965 non-dimensional time units (150 minutes); synchronisation occurs but it is not exact - cell 1 oscillates slightly ahead of cell 2. Concentrations and times are shown in non-dimensional units.

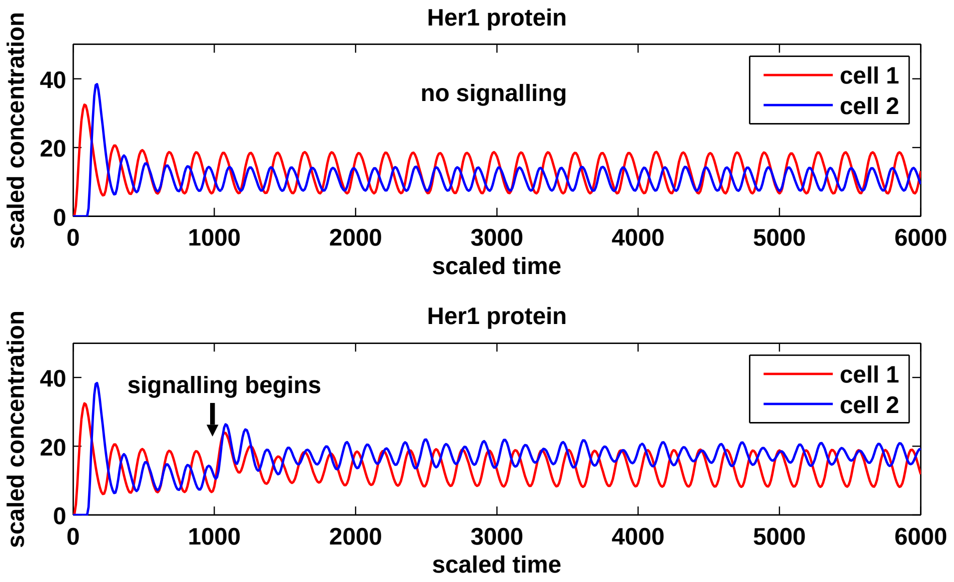

Figure 9: Simulations created in the same way as those in figure 8 except that here the diffusion rates in cell 2 are twice as big as those in cell 1; thus, the non-dimensional diffusion rates are 0.00075 in cell 1 and 0.0015 in cell 2. Signalling does not synchronise the oscillations in the two cells but it clearly influences the dynamics, in particular raising average protein concentrations. Concentrations and times are shown in non-dimensional units.

It follows that (54) and (55) again hold, which we repeat here:

$$m_{\text{ref}} = 1.43 \times 10^{-9}\text{M}, \tag{119}$$

$$k_{\text{her1}} = k_{\text{her7}} = k_{\text{deltaC}} = 1.53 \times 10^{-10}\text{Ms}^{-1}. \tag{120}$$

We suppose that the translation rate of Notch, a_{notch}, is the same as the translation rate of Her1, Her7, and DeltaC. Hence by (118), we have:

$$a_{\text{notch}} = 0.075\text{s}^{-1}. \tag{121}$$

We also suppose the transcription rate of *notch* mRNA, k_{notch}, is the same as the basal transcription rate of *her1*, *her7*, and *deltaC* mRNA. Hence by (120), we have:

$$k_{\text{notch}} = 1.53 \times 10^{-10}\text{Ms}^{-1}. \tag{122}$$

By (111), we know that $k^*_{\text{notch}} = 1$. Using this, and also using (43), (105), and (122), we find that:

$$\text{mnotch}_{\text{ref}} = \frac{k_{\text{notch}}\tau}{k^*_{\text{notch}}} = 1.53 \times 10^{-10}\text{Ms}^{-1} \times 9.33\text{s} = 1.43 \times 10^{-9}\text{M}. \tag{123}$$

By (42) and (110), we know that $a^*_{\text{notch}} = a^* = 1$. Using this, and also using (43), (105), (121), and (123), we find that:

$$\text{pnotch}_{\text{ref}} = \frac{a_{\text{notch}}\text{mnotch}_{\text{ref}}\tau}{a^*_{\text{notch}}} = 0.075\text{s}^{-1} \times 1.43 \times 10^{-9}\text{M} \times 9.33\text{s} = 10^{-9}\text{M}. \tag{124}$$

Given that $\beta^*_1 = \beta^*_2 = \beta^*_3 = 1$ by (111), we can deduce by (106) and (124) that:

$$\text{NICD}_{\text{ref}} = \text{pnotch}_{\text{ref}} = 10^{-9}\text{M}. \tag{125}$$

By (43), (106), (111), and (116), we have

$$\beta = \frac{\beta^*_1 p_{\text{ref}}}{\tau} = \frac{10^{-9}\text{M}}{9.33\text{s}} = 1.07 \times 10^{-10}\text{Ms}^{-1}. \tag{126}$$

Using (107), (111), and (125), we find that:

$$N_0 = N^*_0\text{NICD}_{\text{ref}} = 0.0001 \times 10^{-9}\text{M} = 10^{-13}\text{M}. \tag{127}$$

Using (107), (111), and (124), we find that:

$$M_0 = M^*_0\text{pnotch}_{\text{ref}} = 0.001 \times 10^{-9}\text{M} = 10^{-12}\text{M}. \tag{128}$$

We have now calculated values for all dimensional parameters such that the dimensional Notch signalling model (equations (56) to (74)) will yield oscillatory dynamics in each cell which synchronise. However, we have found that this behaviour occurs for ranges of parameter values. We discuss our method for finding such ranges in the next subsection.

3.3 Parameter ranges yielding synchronised oscillations

Consider the non-dimensionalised Notch signalling model (equations (84) to (102)). If we make the simplifying assumptions in (39) to (41) and in (108) to (110), then the number of model parameters is reduced. The parameters include: D^*, b^*, a^*, k^*, k^*_{notch}, A^*, p^*_{0her}, M^*_0, N^*_0, m, n. If we fix all but one of these parameters as in (42) and (111), then we can vary the parameter that has not been fixed to find a range of its values such that the model yields oscillatory dynamics which

synchronise in the two cells (simulating on the geometry in figure 4 up to $\tilde{t} = 6000$ non-dimensional time units, initial and boundary conditions as in the final paragraph of subsection 3.1, transcription beginning at time 0 in cell 1 and half a period later in cell 2 (that is, at 96.5 non-dimensional time units or 15 minutes), and with signalling beginning at 965 non-dimensional time units or 150 minutes). Conducting a simulation study of this kind for each of the parameters listed earlier in this paragraph allowed us to construct table 2.

Although we will leave a detailed parameter sensitivity analysis for future work, we will note here that, for values of D^* and b^* closer to the extremes of their ranges in table 2, the amplitude of the oscillations becomes smaller and slightly erratic and the synchrony is not quite exact. Moreover, although we state in table 2 that there are synchronised oscillations only when the Hill coefficient m is 1, it is in fact true that synchronised oscillations occur for $m \geq 6$. Yet for $m \geq 6$ the oscillatory period is tripled by the signalling and the amplitudes of the oscillations are increased by a factor of more than 6. Notch signalling is unlikely to have such a big impact on the oscillations, according to experimental studies of the effect of blocking it [2]. Hence we have opted not to include the range $m \geq 6$ in table 2. Further comments on the Hill coefficient m can be found in section 2.3.2 in the paper.

The ranges for the non-dimensional parameters in table 2 can be converted into ranges for dimensional parameters. Assume that the reference time τ, reference length L, and reference concentrations, p_{ref}, m_{ref}, $\text{mnotch}_{\text{ref}}$, $\text{pnotch}_{\text{ref}}$, and NICD_{ref} satisfy (43), (44), (116), (119), (123), (124), and (125) respectively. Then, using equations (20), (32) to (38), (103) to (105), (107), and assumptions (39) to (41) and (108) to (110), we readily obtain by trivial calculations the dimensional parameter ranges in table 2 in the paper.

References

[1] Lewis J (2003) Autoinhibition with transcriptional delay: a simple mechanism for the zebrafish somitogenesis oscillator. Curr Biol 13: 1398-1408.

[2] Ozbudak E, Lewis J (2008) Notch signalling synchronises the zebrafish segmentation clock but is not needed to create somite boundaries. PLoS Genetics 4: e15.

[3] Sturrock M, Terry AJ, Xirodimas DP, Thompson AM, Chaplain MAJ (2011) Spatio-temporal modelling of the Hes1 and p53-Mdm2 intracellular signalling pathways. Journal of Theoretical Biology 273: 15-31 .

[4] Zeiser S, Muller J, Liebscher V (2007) Modeling the Hes1 oscillator. Journal of Computational Biology 14: 984-1000.

[5] Stickney HL, Barresi MJF, Devoto SH (2000) Somite development in zebrafish. Developmental Dynamics 219: 287-303.

[6] Prinz H (2010) Hill coefficients, dose-response curves, and allosteric mechanisms. Journal of Chemical Biology 3: 37-44.

[7] Cinquin O (2007) Repressor dimerization in the zebrafish somitogenesis clock. PLoS Comput Biol 3(2): e32.

**Non-dimensional parameter**	**Range of values giving synchronised oscillations**
Diffusion coefficients D^* (all species)	0.0004 to 0.0023
Degradation rates b^* (all species)	0.008 to 0.036
Translation rates a^* (of Her1, Her7, DeltaC, Notch proteins)	≥ 0.65
Basal transcription rates k^* (of *her1*, *her7*, *deltaC* mRNAs)	≥ 0.49
Basal transcription rate k^*_{notch} (of *notch* mRNA)	≥ 0.000015
Minimal distance A^* of translation from nucleus	0.04 to 0.18
Critical concentration M_0^* of Notch	0 to 10000
Critical concentration N_0^* of NICD	0.0000002 to 0.0012
Critical concentration $p^*_{0\text{her}}$ of Her1 and Her7 proteins	0.04 to 1.71
Hill coefficient m	1
Hill coefficient n	$3, 4$

Table 2: Ranges of parameter values in the non-dimensionalised Notch signalling model (equations (84) to (102)) that yield synchronised oscillatory dynamics. To find each range, we first made the simplifying assumptions on the parameters in (39) to (41) and in (108) to (110). Then we held all but one of the parameters fixed as in (42) and (111) and varied the remaining parameter to find its range of values yielding synchronised oscillations. For each simulation we used the geometry in figure 4, simulated up to $\tilde{t} = 6000$ non-dimensional time units, chose initial and boundary conditions as in the final paragraph of subsection 3.1, let transcription begin at time 0 in cell 1 and half a period later in cell 2 (that is, at 96.5 non-dimensional time units or 15 minutes), and let signalling begin at 965 non-dimensional time units or 150 minutes.
